# A Review on Treatment-Related Brain Changes in Aphasia

**DOI:** 10.1162/nol_a_00019

**Published:** 2020-10-01

**Authors:** Klara Schevenels, Cathy J. Price, Inge Zink, Bert De Smedt, Maaike Vandermosten

**Affiliations:** Experimental Oto-Rhino-Laryngology, Department of Neurosciences, KU Leuven, Leuven, Belgium; Welcome Centre for Human Neuroimaging, Institute of Neurology, University College London, UK; Experimental Oto-Rhino-Laryngology, Department of Neurosciences, KU Leuven, Leuven, Belgium; Parenting and Special Education Research Unit, Faculty of Psychology and Educational Sciences, KU Leuven, Leuven, Belgium; Experimental Oto-Rhino-Laryngology, Department of Neurosciences, KU Leuven, Leuven, Belgium

**Keywords:** aphasia, brain plasticity, neuroimaging, intervention

## Abstract

Numerous studies have investigated brain changes associated with interventions targeting a range of language problems in patients with aphasia. We strive to integrate the results of these studies to examine (1) whether the focus of the intervention (i.e., phonology, semantics, orthography, syntax, or rhythmic-melodic) determines in which brain regions changes occur; and (2a) whether the most consistent changes occur within the language network or outside, and (2b) whether these are related to individual differences in language outcomes. The results of 32 studies with 204 unique patients were considered. Concerning (1), the location of treatment-related changes does not clearly depend on the type of language processing targeted. However, there is some support that rhythmic-melodic training has more impact on the right hemisphere than linguistic training. Concerning (2), we observed that language recovery is not only associated with changes in traditional language-related structures in the left hemisphere and homolog regions in the right hemisphere, but also with more medial and subcortical changes (e.g., precuneus and basal ganglia). Although it is difficult to draw strong conclusions, because there is a lack of systematic large-scale studies on this topic, this review highlights the need for an integrated approach to investigate how language interventions impact on the brain. Future studies need to focus on larger samples preserving subject-specific information (e.g., lesion effects) to cope with the inherent heterogeneity of stroke-induced aphasia. In addition, recovery-related changes in whole-brain connectivity patterns need more investigation to provide a comprehensive neural account of treatment-related brain plasticity and language recovery.

## INTRODUCTION

Aphasia is an acquired neurological language disorder affecting approximately 1 in 250 people (NIDCD, 2015). This is most commonly caused by a cerebrovascular accident in the language-dominant hemisphere, which is the left hemisphere in more than 90% of right-handed persons (Rasmussen & Milner, 1977). Aphasia results in impaired production and/or impaired comprehension of speech, reading, and/or writing. These communication impairments dramatically affect societal participation and integration, causing a substantial decrease in the quality of life (Dahlberg et al., 2006). Effective language treatment might be a crucial element to trigger recovery. Different types of interventions can be used to target the language problems of people with aphasia (PWA). Depending on the affected speech and language components, patients are trained on the production and/or comprehension of the meaning of words and sentences (semantics), the sound structure of words (phonology), written word forms (orthography), grammar (morphosyntax), and/or the melodic intonation patterns inherent to language (melody/rhythm). Other interventions tap communication as a whole (e.g., constraint-induced aphasia therapy; Pulvermüller et al., 2001) and/or social activities and societal participation (e.g., script training; Kaye & Cherney, 2016).

In the last two decades, research on the effectiveness of these therapies has increased, and has provided evidence that high dose speech-language therapy results in better functional communication and better language comprehension and production compared with no intervention. However, effect sizes are weak, inconsistent, and not necessarily evident at follow-up (Brady, Kelly, Godwin, Enderby, & Campbell, 2016). The effects of therapy have also been observed in structural and functional alterations in the brain. It is known that experience, learning, and training can strengthen synapses through the frequent sequential coactivation of connected neuronal assemblies (Tsumoto, 1992). This can consequently alter brain structure and functioning in younger (Scholz, Klein, Behrens, & Johnsen-Berg, 2009) as well as older (Boyke, Driemeyer, Gaser, Buchel, & May, 2008) healthy adults. Varley (2011), who translated known neuroscience principles to aphasia therapy, described that such neuroplasticity should occur after stroke when appropriate interventions, focusing on specific language behaviors, are provided with a sufficient dose, frequency, and intensity (Pulvermüller & Berthier, 2008). Recovery is achieved by either maximizing the capacity of a damaged neural language network or by linking new neural processing assemblies to fulfill a linguistic task (Murphy & Corbett, 2009).

A central question is which regions are considered to belong to “the neural language network.” For centuries, research has been conducted on investigating the neurobiological basis of language. The familiarity of Broca’s and Wernicke’s regions in the context of language is the result of the classic view on linguistic processing proposed by the “Broca–Wernicke–Lichtheim–Geschwind model” in the late 19th century (Geschwind, 1965/2010, 1965, 1970). According to this model, language is situated in the perisylvian area of the left hemisphere, more specifically in the middle and posterior superior temporal lobe for language comprehension, and in the inferior frontal lobe for language production. The connection between Broca’s and Wernicke’s regions is established by the well-known white matter pathway, the arcuate fasciculus (AF; Hagoort, 2014). However, there is still no clear and consistent definition of either of these two regions in terms of anatomical localization. Over the years, Wernicke’s area has been located in almost every part of the posterior perisylvian cortex, including the superior temporal gyrus (STG), the middle temporal gyrus (MTG), and the inferior parietal cortex. In an online survey of specialists in the neurobiology of language, none of the seven anatomical definitions of Wernicke’s region garnered more than 30% of the votes. In addition, in the same survey, only 50% of respondents agreed on the precise location of Broca’s region in the triangular and opercular part of the left inferior frontal gyrus (IFG; Tremblay & Dick, 2016). Furthermore, the strict functional division between language production in Broca’s region and language comprehension in Wernicke’s region is not valid because many fMRI studies have demonstrated that both language modalities share neural resources (Menenti, Gierhan, Segaert, & Hagoort, 2011; Segaert, Menenti, Weber, Petersson, & Hagoort, 2012; Stokes, Venezia, & Hickok, 2019).

In the last two decades, many alternative models for language have been proposed, of which the dual-stream model for speech processing of Hickok and Poeppel (2007) is particularly well known. In this model, multiple regions in the perisylvian cortex, as well as a premotor region and more ventrally located areas, are assumed to underlie linguistic processing. More specifically, the first step in speech perception, the spectrotemporal analysis of the sounds, is assigned to the dorsal STG. Subsequently, phonological processing takes place in the mid-post superior temporal sulcus (STS). The model then proposes a left dorsal stream, underlying the mapping of phonological representations onto articulatory representations, in the parietotemporal junction, the posterior IFG, and a more dorsal premotor region. A ventral stream underlies the mapping of phonological representations onto meaning. Bilateral posterior regions in the ventral stream (posterior MTG and inferior temporal gyrus [ITG]) engage more in lexical semantics, whereas the left anterior regions of the ventral stream are also engaged in sentence level processing (Hickok & Poeppel, 2007).

Other recent language models have proposed an even more extended network, including medial and subcortical structures involved in sensory, motor, and higher-order cognitive processes that support linguistic functioning (e.g., Price, 2000, 2012; Price, Seghier, & Leff, 2010; Vigneau et al., 2006, 2011). The specific role of each of these regions according to these more elaborate language models is provided in Table 1 in the online supporting information located at https://www.mitpressjournals.org/doi/suppl/10.1162/nol_a_00019. Over time, various research groups have studied the healthy neural language network, and different subnetworks have been detected to support different linguistic (semantics, phonology, syntax, orthography) and rhythmic-melodic levels of language (Friederici, 2011; Price, 2000, 2010, 2012; Vigneau et al., 2006, 2011). Yet little is known on treatment-related brain changes in these networks in PWA.

### Aims of the Present Study

In this review, we strive to summarize and integrate the results of recent research on the structural and/or functional changes associated with different language treatments in PWA. The aim is twofold, namely to investigate whether brain changes are (1) specific to the type of intervention received (i.e., phonological, semantic, orthographic, syntactic, or rhythmic-melodic); and (2a) whether the most consistent changes occur within the language network or outside, and (2b) whether these are related to individual differences in language outcomes. Concerning the first aim, we first discuss whether the linguistic interventions (i.e., phonology, semantics, orthography, or syntax) result in specific or similar brain changes. Next, we discuss whether nonlinguistic interventions focusing on rhythmic-melodic aspects rely more on the right hemisphere than linguistic-based interventions. As indicated in the model of Hickok and Poeppel, as well as in Table 1 in the online supporting information, different brain correlates have been related to each of the linguistic components (Hickok & Poeppel, 2004, 2007; Price, 2000, 2010, 2012). It is therefore plausible that, depending on the intervention, different subnetworks undergo changes over time. These differences in intervention effects are assumed to be partly responsible for the extreme heterogeneity in recovery patterns seen in PWA (Saur & Hartwigsen, 2012). However, at the same time, these linguistic components are highly interwoven. This makes it unlikely that only one kind of language processing is tapped during an intervention (e.g., one cannot train sentence production without involving the meaning of the sentence). In addition, there is considerable overlap in the neural networks for different linguistic components (see e.g., Vigneau et al., 2006) and each network presumably interacts with others to create our general language behavior.

**Figure 1.**
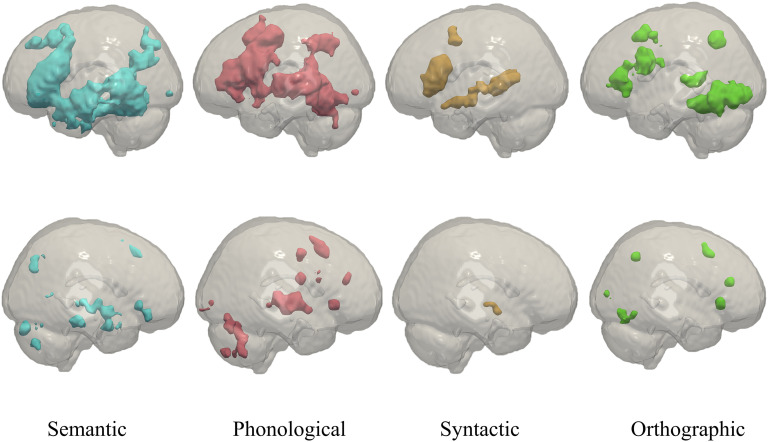
Association test maps for the keywords “semantic” (blue, 1,031 studies), “phonological” (red, 377 studies), “syntactic” (orange, 169 studies), and “orthographic” (green, 132 studies) processing, according to the meta-analysis of Neurosynth (http://neurosynth.org/). First row: left view, second row: right view. The figures were composed using Paraview software (version 5.4.1; https://www.paraview.org/) following the guidelines specified in Madan (2015).

To illustrate this, we overlaid the fMRI association test maps for semantic (blue), phonological (red), syntactic (orange), as well as orthographic (green) processing in Figure 1, based on the automated meta-analysis of previous fMRI studies provided by Neurosynth (http://neurosynth.org/). The association maps represent brain regions where blood oxygen level dependent (BOLD) changes occur more consistently for studies including the search term, than for studies that do not mention the search term. According to Neurosynth, the overlap is the greatest in the left frontal and temporal lobe, with most of the phonological network (red) located dorsally, most of the semantic network (blue) located ventrally, and most of the orthographic network (green) located ventrally and posterior to the other language networks.

Based on these Neurosynth maps, we expected that (1) brain changes related to interventions focused on *phonological* processing are more frequently located in the phonological network (red), (2) brain changes related to interventions focused on *semantic* processing are more frequently located in the semantic network (blue), (3) brain changes related to interventions on *orthographic* processing are more frequently located in the orthographic network (green), and (4) brain changes related to interventions focused on *syntactic* processing are more frequently located in the syntactic network (orange). For all these language interventions, we expected a left-dominance of treatment-related changes. This does not imply that the right hemisphere is not involved in linguistic processing (e.g., see the bilateral ventral stream in Hickok & Poeppel, 2007), but only suggests that it does so to a lesser extent than the left hemisphere.

The rhythmic-melodic network is not depicted in Figure 1, because there was no meta-analysis available on Neurosynth for this keyword. However, it similarly involves brain regions in frontal and temporal lobes (see Table 1 and Figure 1 in the online supporting information), but it is generally considered the only component relying more on the right hemisphere than on the left hemisphere (Baum & Pell, 1999). Thus, we expected more reorganization in the right hemisphere after an intervention on the musical elements of speech, compared with the other linguistic components (i.e., phonology, semantics, orthography, and syntax), which preferentially target the left hemisphere. We also compared the neural effects of interventions targeting the right versus the left hemisphere. Our hypothesis was that brain changes related to interventions focused on rhythmic-melodic processing are more frequently located in frontal and temporal lobes of the right hemisphere.

Concerning the second goal, we first describe whether the most consistent treatment-related changes across therapies occur within the language network (i.e., a combination of response maps visualized in Figure 1) or outside (aim 2a). Although the focus of research on brain–language relations in aphasia recovery has been language centered, Cahana-Amitay and Albert (2015) argued in their review that other cognitive functions, for example, attention, short-term memory, and cognitive control, also contribute to aphasia recovery. In addition, more recent models on the neurobiology of language also have considered brain regions that are involved in multiple other functions (Price, 2000, 2010, 2012). Therefore, we hypothesized that the observed treatment-related brain changes are not restricted to regions classically associated with linguistic processing, but involve a variety of brain structures associated with nonlinguistic cognitive functions.

In addition, we explored whether and how the most consistent treatment-related changes are associated with individual differences in language outcomes (aim 2b). In accordance with findings in Saur et al. (2006), we expected that, at least in the chronic phase post-stroke, normalization of activity to the left hemisphere is most associated with language improvement (restoration). However, because in patients with extensive lesions the left-hemispheric recovery potential is limited, associations between language improvement and brain changes in right-hemispheric regions are expected as well (compensation). For the second aim, we considered treatment-related regional changes as well as changes in connectivity patterns.

## REVIEW METHOD

### Inclusion and Exclusion Criteria

We searched three databases: Pubmed (https://pubmed.ncbi.nlm.nih.gov/), Embase (https://www.embase.com), and Web of Science (https://www.webofknowledge.com) for studies exploring neuroanatomical and/or functional changes in patients with aphasia due to specific language interventions, published between January 2000 and April 2018. More specifically, in the keywords, we combined three main concepts, that is, (1) aphasia, (2) brain changes, and (3) intervention, using different words for each concept (Table 1). If all three concepts were present in the title and/or the abstract, the article was included for further consideration. By screening the reference list of the so-collected articles, relevant papers published after 2000 were additionally added.

**Table 1.** Keyword combinations used to search for intervention studies in patients with aphasia

Concept 1	Concept 2	Concept 3
aphasia	brain changes	intervention
conduction aphasia	brain plasticity	therapy
transcortical sensory aphasia	neural changes	treatment
wernicke	imaging	training
broca	plastic change*	computer-assisted therapy
transcortical motor aphasia	neuroimaging	treatment-induced
anomia	anatom*	rehabilit*
dysphasia	neurobiolog*	
language impairment	neural reorganization	
language disorder		

*Note*. The asterisks represent wildcards and can be replaced by one or more characters (e.g., the search term anatom* will look for terms anatomical, anatomy, etc.).

**Figure 2.**
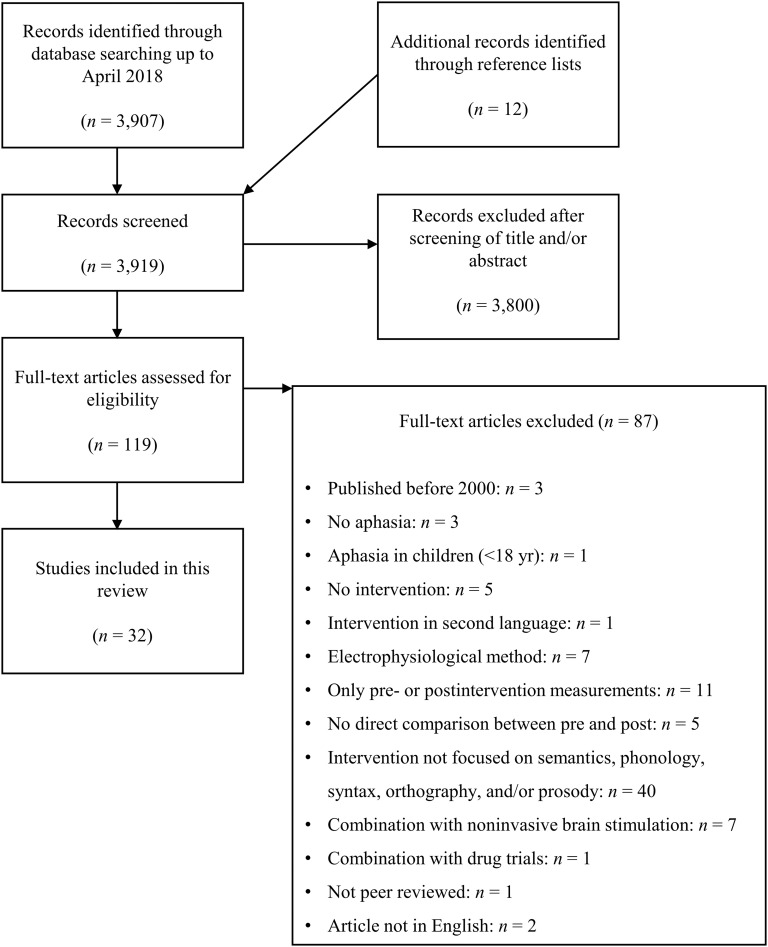
PRISMA flowchart representing the literature search for studies of brain changes in patients with aphasia.

We established the following inclusion criteria. First, the patients had to be adults (to ensure that language and brain development were complete), who had been diagnosed with aphasia as a consequence of a cerebral vascular accident (i.e., stroke). Second, the study had to statistically evaluate the effect of the treatment using measures collected through functional or structural neuroimaging. We decided not to exclude studies on the basis of MRI modality, given that training-induced neuroplasticity can be reflected in functional cortical changes as well as structural white matter changes, and that changes in each modality are related to each other (Honey et al., 2009). Third, each therapy investigated had to focus specifically on one, maximally two, linguistic domain(s): semantics, phonology, syntax, orthography, and/or melody/rhythm, to enable the identification of brain changes after training of these specific types of language processing (aim 1). For this reason, studies providing mixed conventional therapy (e.g., Aerts et al., 2015), intention treatment (e.g., Benjamin et al., 2014), action observation treatment (e.g., Gili et al., 2017), interventions on the activity/participation level, imitation therapy (e.g., Santhanam, Duncan, & Small, 2018), script training (e.g., Fridriksson, Hubbard, et al., 2012), or constraint-induced language therapy (e.g., McKinnon et al., 2017) were not considered. We also excluded studies that combined language therapy with noninvasive brain stimulation and/or drug trials, intervention studies in bilingual aphasia, non-peer-reviewed reports, and studies that were not available in English. Figure 2 represents the literature search process.

### Characteristics of the Studies Included

In total, we identified 32 studies on treatment-related brain changes in PWA that met the inclusion criteria. All references are listed per imaging modality, in-scanner task, and targeted linguistic component in Table 2. For the sake of brevity, we refer to Appendix A for details on each study and to the Supplementary Information for in-depth information on the specific interventions that each study used. (Both can be found in the online supporting information for this article.)

**Table 2.** Selected studies of treatment-related brain changes in patients with aphasia, with applied imaging modality, in-scanner task, and the linguistic component that is targeted during the intervention

No.	Study	Modality	In-scanner task	Targeted linguistic component
1	Abel, Weiller, Huber, and Willmes (2014)	fMRI	ON	semantics (sem, *N* = 9)
2	Cornelissen et al. (2003)	MEG	ON	
3	Fridriksson et al. (2007)	fMRI	ON	
4	Kiran, Meier, Kapse, and Glynn (2015)	fMRI	ON, SFV	
5	Marcotte and Ansaldo (2010)	fMRI	ON, VN	
6	Marcotte, Perlbarg, Marrelec, Benali, and Ansaldo (2013)	fMRI	ON	
7, 8	van Hees, McMahon, Angwin, de Zubicaray, and Copland (2014), van Hees et al. (2014b)	rs-fMRI	ON	
9	Sandberg, Bohland, and Kiran (2015)	fMRI	WJ	
1	Abel et al. (2014)	fMRI	ON	phonology (phon, *N* = 13)
10	Brownsett et al. (2014)	fMRI	SL, SR	
3	Fridriksson et al. (2007)	fMRI	ON	
11	Fridriksson, Morrow-Odom, Moser, Fridriksson, and Baylis (2006)	fMRI	ON	
12	Haldin et al. (2018)	fMRI	RD, SYR	
13	Leonard et al. (2015)	fMRI	SJ, RJ	
14	Marcotte et al. (2018)	fMRI	ON	
15	Rochon et al. (2010)	fMRI	SJ, RJ	
7, 8	van Hees et al. (2014a, 2014b)	rs-fMRI	ON	
16, 17	Vitali et al. (2007, 2010)	fMRI	ON	
18	Nardo, Holland, Leff, Price, and Crinion (2017)	fMRI	ON	
1, 19	Abel et al. (2014, 2015)	fMRI	ON	semantics-phonology (sem+phon, *N* = 6)
20	Fridriksson (2010)	fMRI	ON	
21	Fridriksson, Richardson, et al. (2012)	fMRI	ON	
8, 22	van Hees et al. (2014a, 2014b)	rs-fMRI, DWI	NA	
23	Menke et al. (2009)	fMRI	ON	phonology-orthography (phon+orth, *N* = 2)
25	Raboyeau et al. (2008)	PET	ON	
25	Thompson, den Ouden, Bonakdarpour, Garibaldi, and Parrish (2010)	fMRI	SPM	semantics-syntax (syntax, *N* = 3)
26	Thompson, Riley, den Ouden, Meltzer-Asscher, and Lukic (2013)	fMRI	VN	
27	Wierenga et al. (2006)	fMRI	SG	
28	Jungblut, Huber, Mais, and Schnitker (2014)	fMRI	RCV	rhythm-melody (r − m, *N* = 5)
29	Schlaug, Marchina, and Norton (2008)	fMRI	RSW	
30	Schlaug, Marchina, and Norton (2009)	DWI	NA	
31	Tabei et al. (2016)	fMRI	ON	
32	Wan et al. (2014)	DWI	NA	

*Note*. Some studies target multiple linguistic components and are therefore repeated. For example, several of the identified neuroimaging studies provide a semantic treatment alternated with a phonological treatment. However, they do not always differentiate between the two types of intervention when reporting BOLD changes or they additionally report general BOLD changes over the course of both treatments. The results of these kinds of studies are therefore listed under sem (separate results for the semantic treatment), phon (separate results for the phonological treatment), and sem+phon (mixed results after both treatments). rs-fMRI = resting-state fMRI, DWI = diffusion-weighted imaging, PET = positron emission tomography, ON = object naming, SFV = semantic feature verification, VN = verb naming, WJ = word judgment, SL = sentence listening, SR = sentence repetition, RD = rhyme detection, SYR = syllable repetition, SJ = semantic judgment, RJ = rhyme judgment, SPM = sentence-picture matching, SG = sentence generation, RCV = repetition of chanted vowel changes, RSW = repetition of spoken/sung words, NA = not applicable, BOLD = blood oxygen level dependent.

**Figure 3.**
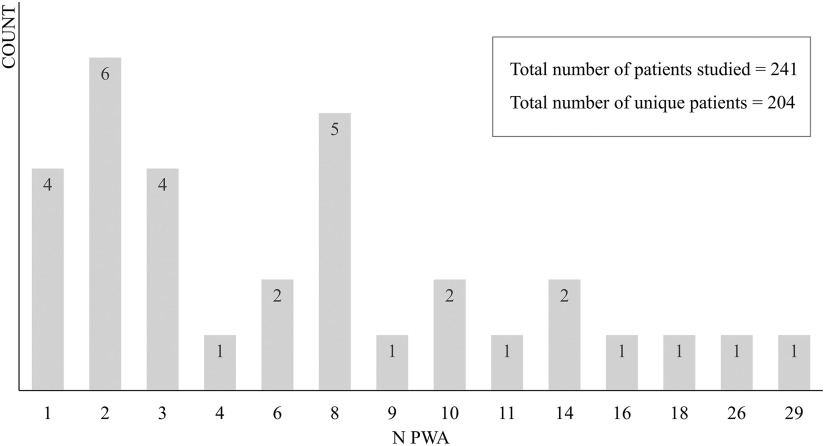
The number of studies (counts) with numbers of participants. PWA = patients with aphasia.

No specific constraint was set on the time post-stroke, but 94% of the studies (30 out of 32) included only patients who were in the chronic stage (≥6 months) post-stroke, to avoid the confounding effects from spontaneous recovery. The remaining two studies included PWA who were at least 4 months post-stroke. Figure 3 shows the number of studies (counts, on the *y*-axis) with different numbers of participants (PWA, on the *x*-axis). In total, 11 studies shared participants with one or two other studies. Seven of these studies were considered separately because they applied different, and mostly unrelated, analyses, that is, task-based fMRI versus resting-state fMRI versus diffusion-weighted imaging (DWI; van Hees et al., 2014; van Hees et al., 2014a, 2014b), voxel-wise whole-brain contrast analysis versus region of interest (ROI) based effective connectivity analysis (Vitali et al., 2007; Vitali et al., 2010) and univariate versus multivariate fMRI analysis (Fridriksson, 2010; Fridriksson, Richardson, et al., 2012). Two studies were considered separately because there was only minimal overlap in participants and different interventions were considered (Fridriksson, Morrow-Odom, Moser, Fridriksson, & Bayliss, 2006; Fridriksson et al., 2007). Finally, the studies of Abel, Weiller, Huber, and Willmes (2014) and Abel, Weiller, Huber, Willmes, and Specht (2015) were considered as one study in this review, because the same voxel-wise whole-brain contrast analysis has been reported in both studies. More details for each study concerning the rationale behind these decisions can be found in the online supporting information. In total, 204 unique patients with aphasia were tested in the articles within the scope of this review.

### Variability Across Included Studies

#### Differences across studies in method, participants, modality, task, and contrasts

To collect a sufficient number of studies on the topic of treatment-related brain changes in PWA, we included both studies reporting standard spatial coordinates for brain regions showing treatment-related neural plasticity and studies that only reported anatomical labels for these regions. For our analysis, we used the anatomical labeling according to the AAL-VOI atlas (Tzourio-Mazoyer et al., 2002). If the study reported standard stereotaxic coordinates, the anatomical labels for these coordinates were derived from the atlas. If the study did not report standard stereotaxic coordinates, the anatomical labels of the study were adopted if the labels corresponded to one of the labels in the atlas. If studies reported labels that did not correspond to a label in the atlas (e.g., [dorsolateral] prefrontal cortex), we assigned the region to one of the labels in the atlas where possible (e.g., middle frontal gyrus), or reported the labels in addition to the atlas labels (which was the case for the inferior frontal sulcus and the premotor area). There are two disadvantages to this choice. First, this review is descriptive because an insufficient number of studies reported standard spatial coordinates to support a quantitative meta-analysis. This, in combination with our choice to focus only on intervention studies targeting maximally two linguistic domains, restricted the number of studies for a meta-analysis. Second, the spatial resolution is reduced because it was not always possible to be sure which parts of an anatomical label were being referred to.

Even when studies used the same imaging modality (e.g., DWI or fMRI), there were substantial differences in methodology (for review, see Crosson et al., 2007). These include differences in fMRI tasks, research design (group study vs. multiple single-subject study), or analysis method (whole-brain vs. ROI analysis). Functional neuroimaging was used in 29 papers to investigate treatment-related changes in the brain, of which 26 applied task-based fMRI, one acquired resting-state fMRI, one applied positron emission tomography (PET), and one applied MEG used with MRI for source localization. Of these functional neuroimaging studies, 62% explored functional changes during an object-naming task (18 out of 29), and five of the included studies additionally concentrated on treatment-related changes in neural connectivity patterns. Finally, three papers applied a structural neuroimaging method, that is, DWI, investigating either local (one study) or distributed white matter changes (two studies). Although Siegel, Ramsey, et al. (2016) demonstrated in a cohort of 132 stroke patients that language relied on both highly localized brain regions, as well as on bilateral brain networks and their connections, surprisingly, in total only seven studies focused on treatment-related changes in neural connectivity patterns (Kiran, Meier, Kapse, & Glynn, 2015; Marcotte et al., 2013; Sandberg, Bohland, & Kiran, 2015; Schlaug, Marchina, & Norton, 2009; van Hees et al., 2014a, 2014b; Vitali et al., 2010). Importantly, five of the seven connectivity studies used an ROI-approach in which the ROIs were chosen based on previous literature or a healthy control group (Marcotte et al., 2013; van Hees et al., 2014b; Vitali et al., 2010; Schlaug et al., 2009). This might have induced bias towards the language network.

When comparing different functional imaging studies, the contrast used to identify a neural response pattern will naturally determine the voxels associated with a specific condition-dependent effect. Some studies applied lenient contrasts, for example, overt picture naming versus rest (e.g., Cornelissen et al., 2003), while others applied very stringent contrasts, such as overt picture naming versus saying “baba” to digitally distorted nonsense images (e.g., Marcotte & Ansaldo, 2010; Marcotte et al., 2012). This makes comparison of studies difficult, because the use of a lenient contrast will identify brain regions involved in a very wide range of processing (from lower to higher level). In addition, across studies, the statistical threshold applied to these contrasts of interest varied. This threshold was sometimes not reported (Marcotte & Ansaldo, 2010; Vitali et al., 2007), and frequently, it was not corrected for multiple comparisons (Abel et al., 2014, 2015; Haldin et al., 2018; Kiran et al., 2015; Marcotte et al., 2013, 2018; Menke et al., 2009; Thompson, Riley, den Ouden, Meltzer-Asscher, & Lukic, 2013; Vitali et al., 2010; Wan et al., 2014). This again complicates the comparison of response foci across studies.

#### Correct versus incorrect language behavior

The studies included differ in whether they included incorrect and/or absent language behavior in their analysis or not (see Appendix A, column “extra,” in the online supporting information). Some studies included all responses in their analysis (correct, incorrect, and no-response items), while others contrasted trained items with correct items pretreatment (trained items > correctly named items pretreatment), as well as incorrect items pretreatment (trained items > incorrectly named items pretreatment). In the latter case, we only included the results of the contrast with correct naming pretreatment. This is because it is assumed that incorrect (language) behavior has a different neural signature (Meinzer et al., 2013) and activates an error network in the brain, including for example the anterior cingulate cortex (Stevens, Kiehl, Pearlson, & Calhoun, 2009), rather than the processing of interest (Price, Crinion, & Friston, 2006). However, it is important to note that this creates a source of variability between studies, as not all of them disambiguated correct and incorrect trials in their analyses. (For a more elaborate discussion of this matter, see Crosson et al., 2007 and Meinzer et al., 2013.)

#### Direction of brain change

Some papers found both upregulation and downregulation of neural activity—in the same brain regions—in different subjects who went through the same language intervention. Increased brain activity, in intensity or extent, might reflect the restoration of neural activity in the perilesional language areas, the engagement of homolog language regions, or compensatory strategies that involve brain regions that are not traditionally associated with language (Cabeza, Anderson, Locantore, & McIntosh, 2002). Increases in brain activity could, on the other hand, also point to inefficient use of neural resources or increased effort when performing language tasks (Fridriksson & Morrow, 2005). In contrast, reduced brain activity accompanied by behavioral improvement could represent increased efficiency in the use of regions (Wierenga et al., 2006), consistent with the effect of practice during skill acquisition (for meta-analysis, see Chein & Schneider, 2005). Decreased activity could alternatively point to persistent malfunctioning, disconnection, or missing input due to the brain damage. Likewise, different recovery mechanisms might also occur simultaneously (Abel et al., 2015). Hence, it is important to relate the BOLD changes to behavioral changes, or to compare them with a healthy control group to interpret them correctly. If there is no relation between brain and behavior, the BOLD changes are hard to interpret, and all that can be generated are hypotheses to be tested in future research. For these reasons, we will not make a difference between upregulation and downregulation of neural activity for the fMRI results in this review, but generally refer to “changes” in the BOLD signal. The difference between increases and decreases in BOLD signal will only be considered below in *Association with language outcomes*, where we discuss how the brain changes are related to behavior, and the interpretation of the results.

#### Treatment-related brain changes

A general problem in neuroimaging reviews is the substantial variability in individual brain reorganization patterns—both within and across studies—which makes comparisons between studies very challenging. Throughout the review, we primarily referred to treatment-related brain changes, which encompassed neural plasticity in the language network, in homologous areas of the right hemisphere, or alternatively, the recruitment of supporting neural infrastructure (e.g., due to a strategy change) or brain dynamics related to (changes in) error processing. This choice reflects that, aside from neuroplasticity in the language network in the left and/or right hemisphere, several alternative processes can induce brain changes over treatment. For example, participants could have had different neural recruitment strategies during language processing before the stroke, related to a difference in task strategy. After the stroke, these differences may be further strengthened by the differential impact of the functional and structural lesions on the brain (Thompson, den Ouden, Bonakdarpour, Garibaldi, & Parrish, 2010). Similarly, participants could rely less or more on supporting cognitive processes after versus before the treatment, (e.g., attention, executive control, and responsive inhibition; Kurland, Baldwin, & Tauer, 2010). In addition, behavioral improvement can manifest in different ways: as an increase in correct attempts or as a decrease in overall errors. Both means of recovery can lead to different brain response patterns (e.g., in the error network as explained in the previous section; Raichle et al., 1994). For comparison purposes, we have tried to include as much study-specific information as possible in Appendix A in the online supporting information.

#### Neurosynth

To answer our second research question, whether the most consistent treatment-related changes across therapies occur within the language network or outside, we used Neurosynth association test maps for the different linguistic components as a reference (Figure 1). It should be noted that this analysis combines highly variable studies. The association map represents a *z*-map for a two-way ANOVA testing for an association between the search term and voxel responses. Because a large number of studies contribute to the meta-analysis, it is assumed to provide a good estimate of the specific response patterns (Yarkoni, Poldrack, Nichols, van Essen, & Wager, 2011).

## RESULTS AND DISCUSSION

The first aim of this review was to explore whether treatment effects are dependent on the focus of the therapy. The second aim was to explore (a) whether the brain regions/networks that most often show treatment-related changes are located within the language network or outside, and (b) whether these changes are associated with individual differences in language (improvement). In Appendix A in the online supporting information, the studies addressing local treatment-related brain changes in specific gray or white matter regions are depicted in italic font. The studies addressing distributed treatment-related brain changes in connectivity patterns are depicted in bold. There was an insufficient number of connectivity studies to investigate the first aim (where they need to be split according to the targeted linguistic component) and therefore these studies are only discussed within the second aim.

### Does the Neural Effect Depend on the Focus of the Intervention?

**Figure 4.**
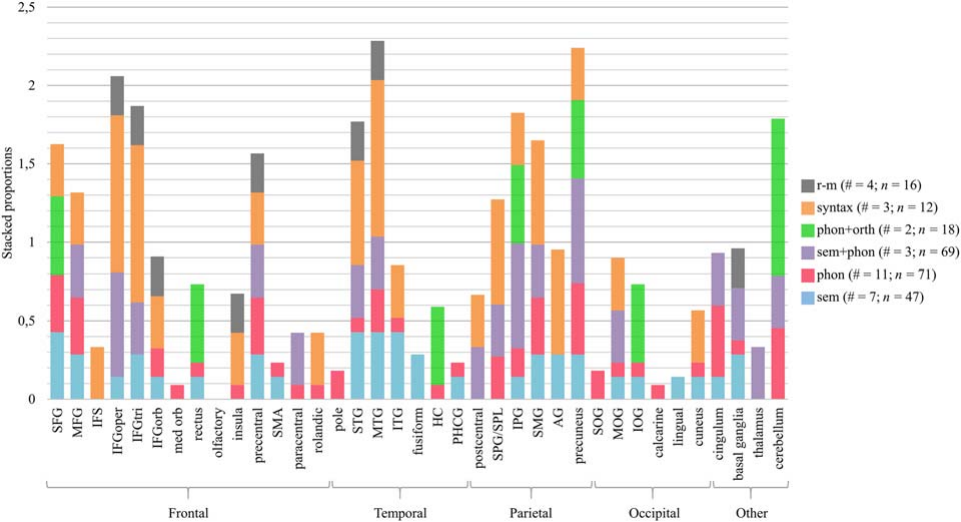
Proportion of studies reporting treatment-related brain changes in a specific brain region of the left hemisphere, relative to the total number of studies providing this type of intervention. The number of studies (#) and patients with aphasia (*n*) per type of intervention is reported in the figure legend. SFG/MFG/IFG = superior/middle/inferior frontal gyrus, IFS = inferior frontal sulcus, oper = opercular, tri = triangular, orb = orbital, med = medial, SMA = supplementary motor area, IPG/SPG = inferior/superior parietal gyrus, SPL = superior parietal lobule, SMG = supramarginal gyrus, AG = angular gyrus, STG/MTG/ITG = superior/middle/inferior temporal gyrus, SOG/MOG/IOG = superior/middle/inferior occipital gyrus, HC = hippocampus, PHCG = parahippocampal gyrus. Anatomical labels other than those included in the AAL-VOI atlas used by the included studies that did not report standard brain coordinates are IFS and SPL.

**Figure 5.**
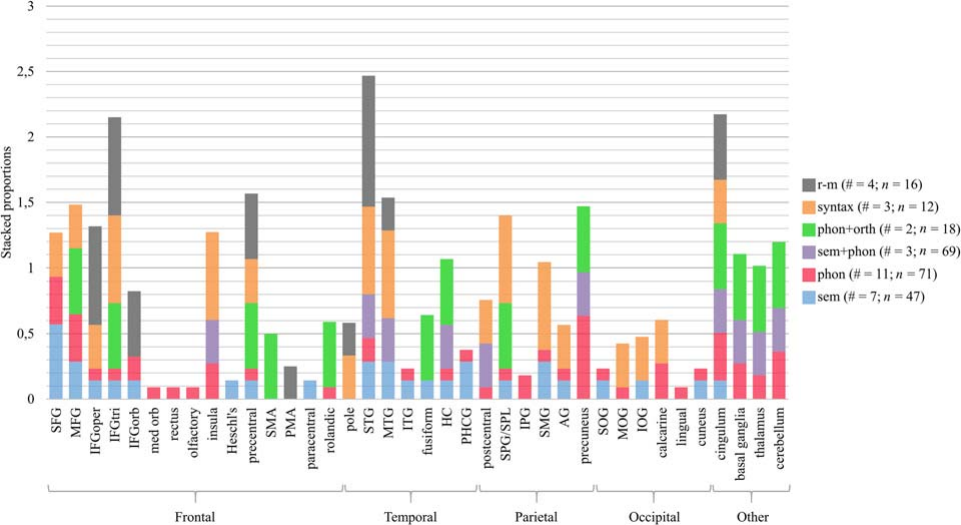
Treatment-related brain changes in the right hemisphere. The number of studies (#) and PWA (*n*) per type of intervention is reported in the legend. Anatomical labels other than those included in the AAL-VOI atlas used by the included studies that did not report standard brain coordinates are PMA and SPL. SFG/MFG/IFG = superior/middle/inferior frontal gyrus, oper = opercular, tri = triangular, orb = orbital, med = medial, PMA = presupplementary motor area, SMA = supplementary motor area, IPG/SPG = inferior/superior parietal gyrus, SPL = superior parietal lobule, SMG = supramarginal gyrus, AG = angular gyrus, STG/MTG/ITG = superior/middle/inferior temporal gyrus, SOG/MOG/IOG = superior/middle/inferior occipital gyrus, HC = hippocampus, PHCG = parahippocampal gyrus.

Figure 4 shows, for all the brain regions in the left hemisphere undergoing intervention-related changes, which types of interventions (sem, phon, sem+phon, phon+orth, syntax, and r-m) have been associated with changes in that area. For example, the left superior frontal gyrus (SFG) was reported in one out of three studies targeting syntactic processing. For this region and this type of intervention, the corresponding proportion is 0.33, which is represented by an orange bar. In addition, the left SFG was reported in four out of 11 studies targeting phonological processing. Therefore, the red bar representing phonological processing has a height of 0.36. This proportion is calculated for every type of intervention, and consequently, for each region, the bar represents a stacked proportion, which can be greater than one. The higher a specific color in the stacked bar, the more the brain changes in that region were specific to the language component. The higher the stacked bar, the higher the number of studies that led to changes in that brain region. In Figure 5 the data for the right hemisphere are represented in a similar way. We also made this figure after excluding studies that did not use a naming task in the scanner (mostly rhythmic-melodic and syntactic processing). As the results are very similar for the other language domains, we will not further discuss this.

#### Neural differences within linguistic interventions

Our first hypothesis was that brain changes related to interventions focused on *phonological* processing are more frequent in regions associated with phonological processing, particularly the left posterior inferior frontal lobe, the dorsal premotor regions, and an area in the parietotemporal junction. Based on Figures 4 and 5 one can see that, although widespread, most brain changes after phonological interventions (red) occurred in the bilateral SFG, middle frontal gyrus (MFG), precuneus, cingulum, and cerebellum, the left supramarginal gyrus (SMG), superior parietal gyrus (SPG), MTG, precentral gyrus, and the right insula, calcarine gyrus, and basal ganglia. Except for the precentral gyrus and the left SMG, these regions are not typically associated with phonological processing. However, the SFG, the cerebellum, the precentral gyrus, and the insula are implicated in motor speech (Ackermann & Riecker, 2010; Stegemöller, 2017; Tourville & Guenther, 2011). PWA, especially the nonfluent subtype, frequently struggle with motor speech planning (Ogar, Slama, Dronkers, Amici, & Gorno-Tempini, 2005) and articulation, which complicates the differentiation of articulatory and phonological errors. Therefore, it could be possible that the phonological language interventions, all targeting speech production, indirectly affected speech-motor processes as well.

Our second hypothesis was that brain changes related to interventions focused on *semantic* processing are more frequent in regions associated with semantic processing, particularly the bilateral posterior MTG and ITG, and the left anterior temporal lobe. As with phonological interventions, therapies focusing on semantic processing (blue) were associated with changes in the bilateral (although left > right) frontal, temporal, and parietal lobes. However, there is also evidence that the temporal lobe was more influenced by semantic interventions, especially in the left hemisphere. More than 40% of semantic studies found brain changes in the left temporal lobe, compared with only 10–25% of phonological studies. Interventions combining *semantic and phonological* processing (purple) led to very mixed results bilaterally (left > right) in the frontal, temporal and parietal lobes, as well as in more medial/subcortical structures and the cerebellum.

The third hypothesis was that brain changes related to interventions involving *orthographic* processing (green) are more frequent in orthographic language networks. Those are situated more posteriorly and ventrally than the previous networks and include the posterior temporal lobe, the fusiform gyrus, the lingual gyrus, the calcarine gyrus, and the cuneus (also see Table 1 in the online supporting information). Again, the results are widespread (right > left), but there was relatively more involvement of the left inferior occipital gyrus (IOG) and the right fusiform gyrus compared with the other interventions. This might be related to the early visual processing of sublexical forms and reading processes that occur in these posterior ventral regions. However, one should keep in mind that these results are based on only two studies.

The fourth hypothesis (mainly based on Vigneau et al., 2006) was that *syntactic* processing (orange) engages inferior frontal regions as well as superior temporal regions, anteriorly as well as posteriorly. Based on Figures 4 and 5 this is clearly the case, although changes were not restricted to these areas. For example, there are also BOLD changes in the bilateral superior parietal lobule and in visual regions of the right hemisphere.

Although we expected that the linguistic interventions would mainly lead to brain changes in the left hemisphere, there is bilateral neural involvement. Due to within-study variation in lesion size and location in different PWA, perilesional activity might have been masked (Crosson et al., 2007). Fridriksson, Richardson, et al. (2012) created patient-specific ROIs of the perilesional cortex and residual naming areas in each lobe. They found that the best predictor of naming improvement was an increase in subject-specific perilesional activity in the frontal areas involved in naming, as well as frontal regions not recruited for naming by the control group. Thus, group studies most probably underestimate the involvement of the left hemisphere in the treatment-related recovery of aphasia. In addition, note that some studies did not show behavioral improvement in (some) participants (see columns “behavioral outcome” and “results” in Appendix A in the online supporting information) and/or included incorrect responses in their analysis (see Appendix A, column “extra”). Therefore, it is possible that some of the results (i.e., the involvement of the bilateral anterior cingulate region, insulae, right parietal lobe, medial temporal lobe, basal ganglia, and thalamus) are related to *error processing* (Stevens et al., 2009). However, most therapies did have a positive behavioral outcome. Another important limitation is that the *amount* of treatment-related brain changes in each brain region was not considered, because these data were not available for every study. This may have masked neural differences in the effect of different types of treatment, since the amount of brain activity (rather than the location) in the affected networks could be specific to the treatment. On the other hand, it could also be possible that treatment-related effects are not specific to the type of therapy administered. In conclusion, treatment-related brain changes do not seem to be very treatment-specific. However, error processing effects and the fact that we were not able to quantify the degree to which brain regions were affected by each treatment, may have masked treatment specificity.

#### Neural differences between linguistic and rhythmic-melodic interventions

Here, we compared interventions targeting the left hemisphere with those targeting the right hemisphere. It is widely accepted that typical linguistic processing is more supported by the left hemisphere than the right hemisphere (e.g., Vigneau et al., 2006), whereas the right hemisphere is more involved in musical, prosodic, and metalinguistic processing (Leon, Rodriguez, & Rosenbek, 2015). More specifically, phonological, semantic, orthographic, and syntactic processing rely more on the left hemisphere. However, there is some involvement of the right temporal lobe in the processing of context, for example during sentence and discourse comprehension, which involves both syntactic and semantic processing (Vigneau et al., 2011). In contrast, there is evidence that linguistic prosody encoded in the intonational contour of a sentence, relies on frontotemporal areas in both hemispheres. The less segmental information, the higher the involvement of the right hemisphere relative to the left one. This functional distinction between the left and right hemisphere begins during acoustic processing in the primary auditory cortex (A1; Friederici, 2011; Witteman, van Ijzendoorn, van de Velde, van Heuven, & Schiller, 2011). According to the asymmetric sampling in the time hypothesis (Poeppel, 2003), left A1 is specialized in processing rapidly changing information with a time resolution of 20–40 ms (e.g., speech sounds), while right A1 prefers the processing of slowly changing information (150–250 ms), such as tonal pitch changes. Based on these findings, we expected that the right hemisphere would be affected by rhythmic-melodic language interventions, such as melodic intonation therapy (MIT) and SIPARI, compared with the other interventions. Similar to MIT, SIPARI combines singing, intonation, prosody, breathing (atmung in German), rhythm, and improvisation (Jungblut, Huber, Mais, & Schnitker, 2014), and therefore places high demands on suprasegmental aspects of language. The initial focus is on vocal training of melodic speech segments assumed to be supported by the right hemisphere. Subsequently, the focus shifts to rhythmic chunking of these speech segments with different complexity levels to stimulate the left hemisphere.

We hypothesized that brain changes related to interventions focused on rhythmic-melodic processing (gray) are more frequent in the frontal and temporal lobes of the right hemisphere. When we compare effects in the left versus the right hemisphere, Figures 4 and 5 show that three out of four r-m studies reported brain changes in the right IFG (in both the triangular and opercular part), while only one out of four studies reported changes in the same structures in the left IFG. Moreover, all r-m studies reported changes in the right STG, while only one study showed changes in its left-hemispheric counterpart. Only in Jungblut et al. (2014), who provided SIPARI-treatment, was there any evidence of left hemisphere involvement. This can be explained by the fact that SIPARI is theoretically structured in such a way that, in addition to the right hemisphere, the left hemisphere is increasingly stimulated over time by shifting the focus from singing to rhythmic chunking of speech. When linguistic and rhythmic-melodic interventions are compared, it can be seen that in the right STG and opercular part of the IFG there are more studies (at least 33%) on rhythmic-melodic interventions that found changes in these regions, than the studies on linguistic interventions. All r-m studies applied a whole-brain contrast analysis or a data-driven ROI-analysis, which precludes bias due to the methodological approach.

#### Conclusions aim one

From the above discussion in *Neural differences within linguistic interventions*, it seems that most brain regions with treatment-related changes were not specific to a particular type of language intervention (because most regions have bars in multiple colors and not one). On the other hand, there are some indications that some regions were more likely to show brain changes when training a specific aspect of language. For example, in the temporal lobe, changes related to a semantic intervention occurred more consistently than changes related to a phonological intervention. Moreover, the studies integrating phonological and orthographic processing led to more changes in the ventral posterior network compared with the other interventions. The interventions focusing on semantic, phonological, orthographic, and syntactic processing seemed to elicit brain changes in both hemispheres. This right-hemispheric involvement in treatments classically targeting the left hemisphere could point to compensatory mechanisms after left-hemispheric brain damage. (For a review see Cocquyt, De Ley, Santens, Van Borsel, & De Letter, 2017.) This right-hemispheric compensation typically takes place in brain regions homologous to language regions in the left hemisphere or in regions involved in more general cognitive functions (e.g., executive functioning; see the next section). From the above discussion in *Neural differences between linguistic and rhythmic-melodic interventions*, it seems that the language interventions focusing on rhythmic-melodic processing included in this review elicited more changes in the right hemisphere compared with the left hemisphere. This right dominance was not found for the linguistic interventions. In general, across treatments and subjects, the regions that are involved in language recovery are very diverse. It is hard to find similar patterns of brain changes between intervention studies targeting the same linguistic component.

### Consistency of Location of Treatment-Related Brain Changes

The second aim of this literature review was to describe whether the most consistent treatment-related changes occur within the language network (i.e., Neurosynth response maps visualized in Figure 1) or outside. We summarized which brain regions showed consistent treatment-related changes across the included studies and investigated whether these ROIs are located within the linguistic maps visualized in Figure 1 (aim 2a). We then explored whether and how these consistent brain changes are related to (a change in) language behavior (aim 2b).

#### Which brain regions show treatment-related brain changes?

Table 3 summarizes which brain regions show treatment-related brain changes across the studies included in this review and how frequently each region is identified. Table 1 in the online supporting information indicates which type of linguistic functions (semantics, phonology, orthography, syntax, and/or rhythmic-melodic processing) have been associated with each of these areas.

**Table 3.** Summary of brain regions showing treatment-related brain changes in patients with aphasia across studies

Change	Frontal	Temporal	Parietal	Occipital	Other*
Left hemisphere	SFG (9)	MTG (11)	SMG (9)	MOG (4)	cerebellum (8)
	MFG (8)	STG (8)	precuneus (9)	cuneus (3)	cingulum (6)
	precentral (8)	ITG (5)	IPG (7)	IOG (3)	basal ganglia (5)
	IFG_oper_ (7)	fusiform (2)	SPG (6)	SOG (2)	thalamus (1)
	IFG_tri_ (7)	HC (2)	AG (4)	lingual (1)	
	IFG_orb_ (5)	PHCG (2)	postcentral (2)	calcarine (1)	
	rectus (3)	pole (2)			
	insula (3)				
	paracentral (2)				
	SMA (2)				
	rolandic (2)				
	med orb (1)				
	IFS (1)				
Right hemisphere	SFG (9)	STG (11)	precuneus (8)	calcarine (4)	cingulum (9)
	IFG_tri_ (8)	MTG (6)	SMG (5)	cuneus (2)	cerebellum (6)
	MFG (8)	fusiform (2)	SPG (5)	SOG (2)	basal ganglia (5)
	IFG_oper_ (6)	HC (3)	AG (3)	MOG (2)	thalamus (4)
	insula (6)	PHCG (3)	postcentral (3)	IOG (2)	
	precentral (6)	ITG (2)	IPG (2)	lingual (1)	
	IFG_orb_ (5)	pole (2)			
	rolandic (2)	Heschl’s (1)			
	SMA (1)				
	med orb (1)				
	PMA (1)				
	paracentral (1)				
	rectus (1)				
	olfactory (1)				

*Note*. The number between brackets indicates how many fMRI-studies (out of 25) show treatment-related changes in this brain region. Of the 32 studies included in the review, five exclusively focus on brain connectivity, while 27 studies report pre-, post, or trained-untrained differences in specific brain regions. Of those 27, the studies by Abel et al. (2014) and Abel et al. (2015) were counted as one because they involve the same participants, and the study by Fridriksson, Richardson, et al. (2012) could not be included since it does not report specific anatomical ROIs (only “perilesional area” vs. “residual naming area”). Thus, in total, 25 studies remain. SFG/MFG/IFG = superior/middle/inferior frontal gyrus, IFS = inferior frontal sulcus, oper = opercular, tri = triangular, orb = orbital, med = medial, PMA/SMA = pre-/supplementary motor area, IPG/SPG = inferior/superior parietal gyrus, SMG = supramarginal gyrus, AG = angular gyrus, STG/MTG/ITG = superior/middle/inferior temporal gyrus, SOG/MOG/IOG = superior/middle/inferior occipital gyrus, HC = hippocampus, PHCG = parahippocampal gyrus, mid = middle. * This category includes regions that do not belong to one of the previous categories, such as subcortical, cingular and cerebellar structures.

As shown in Table 3, the brain regions that were most frequently reported showing treatment-related brain changes in PWA, across all kinds of language interventions, are the bilateral SFG, MFG, IFG, precentral gyri, superior STG, MTG, SPG, SMG, precuneus, basal ganglia, cingulum and cerebellum, the left ITG and inferior parietal gyrus, and the right insula. At least five of the 25 studies showed treatment-related brain changes in these ROIs. This choice reflects effects that are present in one out of five of all treatment-related intervention studies in PWA, which is an effect size reported to be much more common than higher effect sizes (Eickhoff et al., 2016). An important remark is that six of the 25 studies considered here used an ROI-approach in their analysis, which might lead to an overrepresentation of “classic” language areas. However, in five of them, the ROIs were chosen based on the results of a precedent whole-brain analysis, which minimizes the possible bias of the analysis choice.

**Figure 6.**
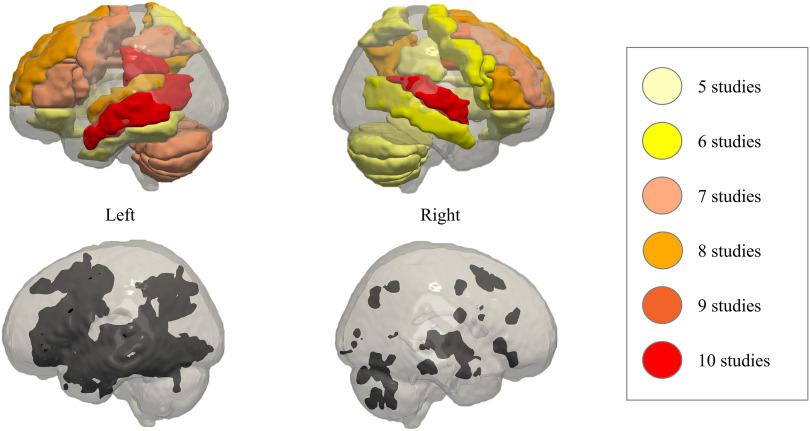
Comparison of Neurosynth language networks (black regions in the lower panel) with regions of interest (ROIs) most frequently associated with treatment-related brain changes (shown on the color map in the upper panel). In the upper panel, the color represents the number of selected studies in which the ROI was reported. In the lower panel, the black regions correspond to association test maps of semantic, phonological, syntactic, and orthographic processing, according to the Neurosynth meta-analysis (http://neurosynth.org/). The figures were composed using Paraview software (version 5.4.1; https://www.paraview.org/) following the guidelines specified in Madan (2015).

The ROIs in Figure 6 represent the regions that were associated with treatment-related neural changes in at least five out of 25 studies (listed in Table 3). As previously explained, for included studies reporting standard neural coordinates, anatomical labels were derived from the AAL-VOIs Single-Subject atlas (Tzourio-Mazoyer et al., 2002). For the studies reporting only ROI labels, their own labels were adopted. This means that a region can be colored if it was reported in (at least) five studies using stereotaxic coordinates, if it was reported in five studies with only anatomical labels, or a combination of both.

Consistent treatment-related changes (five of the 25 studies, in color) overlap with the language network (in black) mainly in the left IFG (the orbital, triangular, and opercular part), the left (ventral) precentral gyrus, the left SMG, IPG and ITG, and in the bilateral STG and MTG. Although the left insula, ITG, fusiform gyrus, and angular gyrus also belong to the traditional language network depicted in black, treatment-related changes have been less consistently found in these regions. Although the language network (in black) is more situated in the left hemisphere than the right hemisphere, treatment-related brain changes (in color) are not restricted to the left hemisphere. Gold and Kertesz (2000) stated that contributions of the right hemisphere are task-dependent and are larger in lexico-semantic processing than phonological processing. This arises because, in the healthy brain, lexico-semantic processing is less left-lateralized (i.e., more bilateral) than phonological processing. However, in Figure 5 it can be observed that all treatments evoked changes in right-hemispheric ROIs, not only interventions focusing on semantic processing. Structures wherein treatment-related brain changes occurred consistently over different types of treatment, and which do not nicely overlap with the linguistic network (in black) were the lateral, orbital as well as medial part of the bilateral superior and middle frontal gyri, the left hippocampus, paracentral lobule and IOG, the bilateral precuneus, cingulate, cerebellum, SPG, and the right basal ganglia. However, for the right cerebellum and the left precuneus, there were some overlapping “dots.” Figure 6 might overrepresent the frontal and cerebellar areas, because we were limited to visualize ROI labels instead of specific MNI-coordinates. Not all studies reported these coordinates, and these ROIs tend to encompass large areas of the brain.

Concerning connectivity, the included functional and effective connectivity studies (not represented in Table 3 or Figure 6) found modulations of the connectome in very similar regions. van Hees et al. (2014b) and Sandberg et al. (2015) demonstrated posttreatment modulations of resting-state and task-related functional connectivity strength, respectively. Upregulation of connectivity strength was found in the language network of PWA in and between both hemispheres, as well as in the bilateral SFG, left MFG, precuneus, and precentral gyrus (Sandberg et al., 2015). On the other hand, there was a downregulation of connectivity between bilateral language regions and the right basal ganglia, cingulum, and cerebellum (van Hees et al., 2014b). Kiran et al. (2015) and Vitali et al. (2010) showed treatment-related changes in task-related effective connectivity patterns. In agreement with the functional connectivity results, effective connectivity modulations existed throughout the language network in and between both hemispheres, as well as between the IFG and the MFG in both hemispheres (Kiran et al., 2015). Furthermore, Marcotte et al. (2013) demonstrated that language interventions in PWA were able to normalize the amount of functional integration within the posterior default mode network (the bilateral MTG, the AG, the left ITG, the right middle cingulate, and the right cerebellum). This network is more active during conscious resting states of the brain compared with during the performance of a cognitive task (Cavanna & Trimble, 2006).

#### Association with language outcomes

We explored whether the measured brain changes in the regions visualized in Figure 6 show (positive) associations with language outcomes. As explained above in *Direction of brain change*, relating the brain changes to behavioral change is necessary to interpret the meaning of the activity patterns. Therefore, in this section, we will consider the difference between an increase and a decrease in the measured neural response over the course of the treatment.

Among the consistently identified ROIs (colored regions in Figure 6) overlapping with the language network, BOLD increases in the left IFG_oper_ (Fridriksson, 2010), BOLD decreases in the left STG, MTG, and SMG (Abel et al., 2014), and BOLD increases as well as decreases in the IPG (Abel et al., 2014; Fridriksson, 2010; Raboyeau et al., 2008) have been related to language improvement across studies. These studies highlight the importance of the left hemisphere for aphasia recovery, although it is not clear why Abel et al. (2014) found BOLD decreases in these regions, in contrast to the other studies. However, they found a negative association between activity decrease and therapy gains, implicating the importance of continued reliance on the left hemisphere during the treatment. For the right hemisphere, there are two main hypotheses concerning its involvement in the post-stroke neural response pattern. In the first hypothesis, left-hemisphere damage is thought to induce pathological transcallosal disinhibition of the right-hemisphere homologs. As such, the proponents of this presupposition, view right-hemispheric activity as detrimental (e.g., Heiss & Thiel, 2006; Price & Crinion, 2005; Saur et al., 2006). Support for this hypothesis comes from the positive association between language improvement and BOLD decreases in the right insula and IFG_oper_ (Nardo et al., 2017), the right precentral gyrus and precuneus (Raboyeau et al., 2008), or a decrease in fractional anisotropy of the IFG_oper_ (Wan et al., 2014). However, two other studies in this review found positive associations between language improvement and brain responses in different right hemisphere regions (MFG, SMA, fusiform gyrus, hippocampus, SPG, putamen and anterior cingulate). This suggests that the right hemisphere could also support language recovery in the chronic phase after stroke (Menke et al., 2009; Raboyeau et al., 2008).

Positive correlations between language improvement (picture naming or picture description) and response (changes), in regions that are not traditionally associated with language, suggest that these structures may play a role in recovery from aphasia, particularly naming. More specifically, there were positive associations between naming improvement and BOLD signal in the bilateral SFG (Brownsett et al., 2014), BOLD increases in the bilateral MFG (Fridriksson, 2010; Raboyeau et al., 2008), BOLD decreases in the bilateral precuneus (Raboyeau et al., 2008), BOLD increases in the left precuneus (Fridriksson, 2010), posttreatment BOLD signal in the right precuneus (van Hees et al., 2014), BOLD decreases in the left paracentral lobule (Abel et al., 2014), BOLD increases in the left IOG, left cerebellum, bilateral hippocampus, and the right SPG (Menke et al., 2009), and BOLD increases in the right putamen and the right anterior cingulate (Raboyeau et al., 2008). In summary, across different studies, changes in left-hemispheric language regions, right-hemispheric homologs, as well as bilateral regions not traditionally associated with language have been associated with language improvement over treatment. It is very likely that part of this variability is caused by variations in lesion size and site, which determines whether there is still recovery potential in the left hemisphere, as well as premorbid lateralization patterns (Warburton, Price, Swinburn, & Wise, 1999) and time post-stroke (Saur et al., 2006).

Among the connectivity studies, Vitali et al. (2010) showed associations of modulations in task-related effective connectivity patterns throughout the language network in and between both hemispheres with correct picture naming of trained items. In contrast, the correlations between the amount of functional integration within the posterior default mode network and naming improvement was not significant in the study by Marcotte et al. (2013). Overall, these results suggest that modulation and normalization of functional and effective connections within and outside the language network are concurrent, but not necessarily correlated, with aphasia recovery. In addition to functional connectivity, recent research has suggested that intact structural connectivity is beneficial for successful aphasia recovery (Bonilha, Gleichgerrcht, Nesland, Rorden, & Fridriksson, 2016; Bonilha et al., 2017; Griffis, Nenert, Allendorfer, & Szaflarski, 2017; Yourganov, Fridriksson, Rorden, Gleichgerrcht, & Bonilha, 2016). Several studies have demonstrated the importance of the AF for language recovery after stroke, especially for improvement in speech production (Breier, Juranek, & Papanicolaou, 2011; Hosomi, Nagakane, & Yamada, 2009; Jang, 2013; Jang & Lee, 2014). This finding is consistent with the assumed function of this white-matter pathway in mapping acoustic representations of sounds with their motor representation (Saur et al., 2008). In the study by van Hees et al. (2014a) included in this review, the pre- and posttreatment mean generalized fractional anisotropy, an indirect measure of fiber bundle characteristics, of the left AF correlated with maintenance of (phonological) treatment gains, and the fractional anisotropy value increased over treatment. This observation complements that of Marchina et al. (2011) and Wang, Marchina, Norton, Wan, and Schlaug (2013) who showed that lesion load of the left AF significantly predicted the level of impairment in language production in chronic stroke patients, explaining more variance in language behavior than the functional gray matter lesion load. It also has been shown that better language performance after irreversible damage in the left hemisphere is associated with increased structural connectivity in the right AF (Forkel et al., 2014). Fridriksson et al. (2006) demonstrated significant naming recovery of a subject sparing some white matter connections in the inferior frontal lobe, while a patient with more extensive white matter damage in this area did not recover. Because the AF is connected to the posterior part of the ventrolateral frontal lobe (Catani, Jones, & Ffytche, 2005), these white matter connections might have been part of it.

#### Conclusions aim two

In summary, across studies, many brain regions have been associated with treatment-related language recovery, although in a very inconsistent way, which makes it difficult to make any decisive conclusions. Although the left IFG, STG, MTG, and inferior parietal regions are typically considered to be involved in linguistic processing (Geschwind, 1970; Hickok & Poeppel, 2007), their right counterparts and the bilateral SFG, MFG, precentral gyri, SPG, precuneus, cerebellum, cingulum, right insula, and basal ganglia are not. The latter regions are mainly known for their nonlinguistic functions. Thus, it is possible that brain regions more medial and/or subcortical to what is generally studied in the context of language processing, are additionally involved in the process of aphasia recovery. In addition, a variety of other cognitive functions have been attributed to the brain regions that are typically considered to be involved in language processing (Geschwind, 1970; Hickok & Poeppel, 2007), such as feedback mechanisms (motor and auditory); the planning, coordination, timing, execution, and control of (speech) movements; amodal semantic processing; learning; attention; and other higher-order executive functions.

Language improvement is evident not only in changes in distinct gray matter regions, but also in their functional connectivity patterns and in the white matter pathways enabling direct communication between these regions. More research on the role of brain connectivity in aphasia recovery is necessary to fully understand functional deficits beyond the lesion and to evaluate reorganization potential after stroke at the neural network level. Methodological shortcomings and variability between studies (e.g., in lesion size), as well as the large inconsistency in results across the different studies make it hard to present clear conclusions on treatment-related brain changes in PWA. We cannot put forward that language regions show consistent treatment-related changes, nor can we conclude that other regions involved in cognitive processing show consistent changes. There is not, therefore, more evidence for changes in the language network than for changes outside the language network. In the next section, we will discuss these issues in more detail and formulate suggestions for future research.

### Limitations and Future Directions

Over the past two decades, an increasing number of studies have investigated treatment-related brain changes in PWA after stroke. In this review, we integrated the results in a descriptive way to provide the current state-of-the-art on this topic. We are well aware that even in this descriptive comparison one should be cautious when interpreting the results because of variability between as well as within studies. In this section, we will summarize the limitations that we came across throughout the review and formulate recommendations to enable quantitative and more reliable comparisons in the future. Table 4 provides a short overview of the limitations and recommendations, which will be elaborated on throughout the text.

**Table 4.** Overview of the limitations and recommendations for future work in the area of treatment-related brain changes in patients with aphasia

Topic	Limitation	Recommendations
Methodology	Variability between studies in operationalization of the experiments, neuroimaging pipelines, and statistical analyses	Systematic integration of diverging findings (meta-analyses)
	Lack of reporting standard stereotaxic coordinates	Feport standard stereotaxic coordinates for neural effects
	Combining different lesion patterns	Consider individual data points in the analysis and consider the effect of the lesion
	Potential unreliability of the hemodynamic response	Use patient-specific hemodynamic response functions in modeling of the response
Interpretation of results	Risk for habituation of the patient to the fMRI task	Use multiple pre- and postscan sessions or control fMRI-tasks
	Variability in direction of the change in the BOLD signal	Relate brain changes to behavior and other patient-specific factors
	Focus of treatment studies on the chronic phase post-stroke	Compare findings in chronic phase post-stroke with findings in acute and subacute phase
	Systematic interstudy patient heterogeneity	Avoid selection bias in the study design
Sample size	More than 70% of the studies have a sample size smaller than 10	Perform studies of aphasia recovery in larger samples of patients
Treatment-related effects outside of the language network	Overlap between the language network and several non-language processes	Characterize the function, importance, and language specificity of regions that have been consistently identified in aphasia recovery (whole-brain analyses)
Connectivity studies	Focus on regional changes in functional response patterns	Explore treatment-related brain changes on the connectome level, structurally as well as functionally
Individual variability as meaningful information	Results are collapsed across the subject dimension	Consider data at the individual subject level

#### Methodological limitations

Although the main aim of this research was to gain insight into the mechanisms underlying aphasia recovery, there are considerable differences in the operationalization of the experiments, the neuroimaging pipelines, and the statistical analyses among the included studies. In summary, there are differences in the imaging modality (PET, MEG, fMRI, DWI), research design (group study vs. [multiple] single-subject study), in-scanner language task (targeted linguistic component, production vs. comprehension, overt vs. covert, word vs. sentence level), assessed contrast (lenient vs. stringent, pre-post vs. trained-untrained), neuroimaging analysis approach (whole-brain vs. ROI-approach), modeling of the trials in the statistical model (including vs. excluding incorrect trials, collapsing data from multiple time points or not), and the statistical analysis itself (e.g., *t*-test, *F*-test, [multiple] regression analysis, partial least squares analysis, correlation analysis). The systematic integration of the diverging findings of these studies might overcome this variability to some extent. However, failing to report peak locations of treatment-related effects in stereotaxic reference space makes coordinate-based meta-analysis at present impossible. This is an important limitation and point of concern for future research, because meta-analyses could compensate for the highly variable results from the small-data studies, which are still very common in this field of research (Eickhoff et al., 2016).

Several methodological limitations are related to the brain lesion. To take into account the role of perilesional activity in language recovery over time, it is important to consider individual data points in the analysis (e.g., regression). In group analysis of PWA with diverse lesion patterns, there is a lack of power to detect changes in perilesional areas, even when the group is highly homogeneous (Crosson et al., 2007; Meinzer et al., 2013). This variability in lesion size and location also leads to a lower statistical power in the lesioned left hemisphere as compared with the structurally intact right hemisphere. In particular, the effect of lesion site and size on treatment-related recovery should be evaluated, because it is known to be one of the most predictive factors in aphasia prognosis (e.g., Plowman, Hentz, & Ellis, 2012). However, only a few studies included lesion information as a confounding variable in their correlation or regression analysis (parametrized as lesion volume or IFG lesion load; Brownsett et al., 2014; van Hees et al., 2014a; Wan et al., 2014). As shown by Wang et al. (2013), it might be interesting for future studies to additionally include a measure of AF-lesion load, because this explained more variance in language behavior than functional gray matter lesion load. Only two studies performed a separate analysis to specifically assess the effect of the lesion on recovery patterns (i.e., voxel-based lesion recovery analysis (Fridriksson, 2010) and joint posttreatment independent component analysis (Abel et al., 2015). Crinion, Holland, Copland, Thompson, and Hillis (2013) provided guidelines for the quantification of brain lesions after stroke, since in this area, there is also substantial variability in methodology.

An important limitation and point of concern for future research is the potential unreliability of the hemodynamic response in stroke populations with cerebrovascular damage. This response is dependent on cerebral blood flow, cerebral blood volume, and oxygen consumption. There is evidence that the neurovascular coupling response, which underlies fMRI and is typically modeled by the hemodynamic response function (HRF), is reduced and delayed in stroke patients (Bonakpardour, Parrish, & Thompson, 2007; Crinion & Leff, 2007; Lake, Bazzigaluppi, & Stefanovic, 2016; Thompson et al., 2010) and even in healthy aging (Nair, Raut, & Prabhakaran, 2017). Siegel, Snyder, Ramsey, Shulman, and Corbetta (2016) demonstrated that over one-third of stroke patients show hemodynamic lags two weeks post-stroke, dropping to 15% three months post-stroke and 10% one year post-stroke. Importantly, the amount of lag severity was correlated with lesion size and severity of deficits in multiple domains. Some studies included in the review tried to consider this by estimating patient-specific HRFs using long-trial fMRI, to enhance the detection of BOLD changes in brain regions with delayed HRFs (e.g., Thompson et al., 2010). Moreover, by conducting additional perfusion imaging, hypoperfused tissue could be identified. Indeed, Thompson and colleagues showed associations between decreased blood flow and an increased time-to-peak value of the HRF, and between perfusion levels and treatment-related BOLD changes. Importantly, this hypoperfusion was not limited to the perilesional area, extending the lesion to remote brain regions, even in the contralateral hemisphere, although perfusion values were generally higher there than in the affected hemisphere. Therefore, studies that limit their analysis to a canonical model of the HRF, based on healthy brain responses, might underestimate or completely fail to detect functional activity in affected regions (Bonakdarpour, Beeson, Demarco, & Rapcsak, 2015).

Subject-level analyses allow for more careful consideration of not only the lesion but also differences between PWA in age, gender, education level, time post-stroke, already received intervention, aphasic symptoms, task strategy, and response to treatment (see also *Individual variability as meaningful information* below). All these sources of variability add to the challenge of finding consistent results in aphasia recovery studies. In an ideal situation, insights in pre-stroke brain structure and function would highly improve our understanding of neuroplasticity after stroke. This can only be achieved by conducting large-scale longitudinal studies of people at high risk for stroke, considering factors such as family history, arterial hypertension, hyperlipidemia, diabetes, nicotine, and/or alcohol (ab)use. Another option is to assess undamaged structures immediately after stroke, to be ahead of brain reorganization as much as possible.

#### Interpretation of results

The location of the treatment-related effects in each study is highly dependent on the large number of (subjective) choices on the specific implementation, as listed above. A crucial issue that is not typically addressed in intervention studies of PWA is the distinction between treatment-related brain changes and overall scan-rescan variability, especially the neural effect of task learning (Chein & Schneider, 2005). If PWA habituate to the imaging task from pre- to post-intervention, neural activity in task-related regions will most probably decrease, independent from the effect of the treatment. Three possible options to disentangle the effects of the treatment from practice-related reductions in brain activity (Rapp, Caplan, Edwards, Visch-Brink, & Thompson, 2013) are (a) to compare the fMRI-task of interest with a control task, (b) to compare treated with untreated items, and (c) to estimate the test-retest variability by performing multiple scan sessions pre- and/or posttreatment (Brownsett et al., 2014; Cornelissen et al., 2003; Fridriksson et al., 2007; Fridriksson, 2010; Fridriksson et al., 2006; Fridriksson, Richardson, et al., 2012; Sandberg et al., 2015; Schlaug et al., 2009). However, 11 studies did not perform multiple scan sessions, or mention or use an appropriate control task (such as null events, looking at a fixation cross, or rest; Abel et al., 2014, 2015; Jungblut et al., 2014; Marcotte et al., 2018; Menke et al., 2009; Raboyeau et al., 2008; Tabei et al., 2016; Thompson et al., 2010, 2013; Vitali et al., 2007, 2010), and only one of those 11 contrasted trained items with untrained items to compensate for this (Vitali et al., 2007).

The interpretation of the meaning of the effects is further complicated by the variability in the direction of the change in BOLD response in both hemispheres across PWA and studies, as well as the diverging possible causes of treatment-related neural plasticity. In summary, some of the included studies reported correlations between increased activity in (homologous) right-hemispheric regions and language improvement (Menke et al., 2009; Raboyeau et al., 2008), which is frequently interpreted as compensation. Others reported associations between activity decreases in bilateral brain regions and better language performance, which is attributed to increased task processing efficiency (Nardo et al., 2017; Raboyeau et al., 2008). In contrast, other studies found associations between therapy-induced language improvement and increased (or less decreased) activity in bilateral brain regions, including perilesional and spared left-hemispheric areas. This might be related to increased task demands, successful reorganization patterns or maladaptive plasticity (Abel et al., 2014; Fridriksson, 2010; Menke et al., 2009; Raboyeau et al., 2008). In the future, careful and individual consideration of lesion site and size, treatment strategies, response to treatment, and behavioral symptoms is needed to understand why and how the changes in the BOLD signal are related to behavioral improvement (Hartwigsen & Saur, 2019). Moreover, comparisons with intervention studies in PWA in the subacute phase after stroke are needed to take into account and to understand the dynamic nature of brain plasticity across the time course of recovery (Saur et al., 2006; Saur & Hartwigsen, 2012). At present, intervention studies almost exclusively took place in the chronic phase after stroke, although it is generally assumed that most language recovery takes place in the first days to weeks after stroke, and the mechanisms involved are most probably different.

Another issue that impacts the interpretation of the results concerns the inclusion criteria for the various interventions provided in the different studies. If specific behavioral symptoms were prerequisites to receive a specific treatment, the inclusion criteria of the different studies induced a systematic selection bias that ultimately also would have influenced the (generalizability of the) results. To explore this, we checked the patient-specific inclusion criteria of all included studies and found that, overall, the studies providing anomia treatments (sem, phon, sem+phon and phon+orth interventions) selected patients with at least a moderate naming deficit or did not mention any inclusion criteria. Remarkably, the majority of phonological studies included patients with nonfluent aphasia, while semantic (and sem+phon) studies included patients with various aphasia profiles or even fluent aphasia. The studies providing interventions on the syntactic level all included agrammatic patients with nonfluent aphasia. The studies providing rhythmic-melodic interventions also specifically included patients with (mostly moderate to severe) nonfluent aphasia. Because the difference in aphasia symptoms/profiles across studies may translate to a difference in lesion patterns, this selection bias could have systematically influenced the amount of change that was possible in the different linguistic subnetworks.

#### Sample size

Due to the specific patient population and practical concerns, studies in PWA frequently have to deal with small sample sizes. The sample size of the included studies varies from one to 29 patients, with more than 70% of the studies (23/32) having a sample size smaller than 10. Figure 3 shows the number of studies (on the *y*-axis) including a certain number of PWA (on the *x*-axis). From this figure, it is clear that more than 70% of the included studies of treatment-related changes in PWA include 10 participants or less. As Ramus, Altarelli, Jednoróg, Zhao, and Scotto di Covella (2018) described in their review, neuroimaging studies with such limited sample sizes are statistically underpowered to detect group differences. As a consequence, uncorrected (or not adequately corrected) results are frequently reported, which are more likely to be unreliable (Ramus et al., 2018). Thus, in this case, one problem (underpowered studies) might lead to another (reporting spurious results), preventing the field from moving forward.

In particular, when looking for differences with small to medium effect sizes, which is the case for treatment-related brain changes in PWA, a large sample is needed to detect within-group differences with adequate (not too large) confidence intervals (Ramus et al., 2018). For example, to have a power of 80% to detect small within-subject differences (*η* ^2^ = 0.02) using within-subjects repeated measurements ANOVA (4 measurements), one needs to include at least 69 participants. The number of PWA should be even higher than this to deal with fewer measurements, the large heterogeneity in the aphasia population, for example in lesion pattern and symptomatology, and other confounds mentioned earlier. This number stands in great contrast with the number of participants in the studies included in this review and in studies that are still being published in the field of aphasia recovery, with few of them reaching this standard. It is, therefore, crucial to perform studies of aphasia recovery in larger samples of patients to ensure sufficient statistical power to demonstrate a treatment effect, to allow for generalization to other (sub)populations, and to account for methodological limitations (e.g., smoothing and the need to correct for multiple comparisons; see Poldrack, Mumford, & Nichols, 2011). Because large scale studies in a population of patients of aphasia are practically very challenging, data sharing might be a suitable alternative to increase sample size. We refer the reader to Meyer (2018) and Poldrack and Gorgolewski (2014) for an overview of data-sharing efforts in the field of fMRI and practical tips for ethical data sharing, respectively.

#### Treatment-related effects outside the language networks

Our review has shown that recovery from aphasia engages brain regions that are outside those traditionally associated with language functions (Figure 6). Such effects might relate to other functions (such as cognitive control) that are not specific to language but indirectly support language. It has already been suggested that neural activity in the right IFG, a region that has been consistently found to be involved in language recovery (Turkeltaub, Messing, Norise, & Hamilton, 2011), including in our own review, is more related to top-down cognitive control by a cingulo-opercular network than to a dynamic language-specific process (Geranmayeh, Brownsett, & Wise, 2014). Given the overlap between the language network and several non-language processes, such as cognitive control or episodic memory—see, for example, Chein and Schneider (2005), Geranmayeh, Brownsett, and Wise (2014), and Humphreys and Lambon Ralph (2015)—it seems worthwhile to characterize the function, importance, and language specificity of other regions that have been consistently identified in aphasia recovery, including subcortical brain regions. The relative contribution of these regions to language recovery compared with the perisylvian regions traditionally associated with linguistic processing (Geschwind, 1970; Hickok & Poeppel, 2007) is presumably even more important than those we can derive from the results of this review. Approximately one-third of the included studies applied an ROI analysis, which in half of them, focused on exploring the role of language regions in the left hemisphere and their right-hemispheric counterparts in the response to aphasia interventions. Therefore, we believe that whole-brain analyses are required to fully understand the neural correlates of the cognitive mechanisms that support treatment-related language recovery. Moreover, it might be interesting to further explore the effect of targeting these cognitive functions during language interventions on aphasia recovery (Cahana-Amitay & Albert, 2015). For example, it would be useful to know whether these treatments (e.g., working memory training) are as effective as language interventions at boosting language recovery and whether PWA could benefit from a combination of both.

#### Connectivity studies

Another striking finding is that the majority of the included research (around 80%) focused on regional changes in the brain, while only seven studies investigated treatment-related changes in connectivity patterns. As language and other cognitive functions are linked to large-scale neural networks, formed by interconnected cortical and subcortical areas, stroke-induced damage to that network can lead to distributed dysfunction far beyond the lesion (Carter, Shulman, & Corbetta, 2012). Indeed, experimental animal studies have shown that stroke unavoidably affects the brain connectome within minutes of onset (Silasi & Murphy, 2014). Therefore, treatment-related (or spontaneous) regional alterations should be considered in the context of these brain-wide connections that might explain more of the recovery process in PWA than considering discrete brain regions in isolation. Due to recent advances in neuroimaging and computational sciences, more and more studies could and should explore treatment-related brain changes on the connectome level, structurally as well as functionally. Again, for these kinds of analyses, a large sample size is very important. In addition, analyses on the network level might be more sensitive to reveal distinct effects of interventions targeting different linguistic components. It is very likely that treatments, to some extent, do not differ in the *location* of their effect, but rather in the specific *distribution* of neural activity over the (same) language network, as well as other supporting cognitive networks. In addition, one can investigate functional connectivity patterns using resting-state fMRI, which has several advantages. First, it requires minimal participation, which makes it clinically more interesting compared with task-based fMRI, especially in the case of severe aphasia or in the acute phase post-stroke. Second, the interpretation of functional connectivity is not complicated by different task strategies, as in task-based fMRI (Siegel, Shulman, & Corbetta, 2017). In summary, investigating connectivity patterns offers a better understanding of the effects of the lesion on the networks in the brain (extended lesion effects), as well as a better understanding of how treatment influences the interaction in and between specific distributed networks (language, but also memory, executive function, etc.). We contend that a regional approach and a connectivity approach are complementary methods that together can answer different research questions (i.e., whether we are interested in treatment effects on specific regions or on how they work together to create language).

#### Individual variability as meaningful information

Approximately half of the included studies included a control group of healthy adults, mostly to provide normative data on (repeated) task-specific brain response patterns. The results for the healthy control group are therefore collapsed across the subject dimension and only the mean effects are considered. In the context of personalized medicine, it might be interesting not to treat the between-subject variance in the neuro-anatomical representation of language as noise, but rather as meaningful information. After all, interindividual variability in a healthy population (e.g., differences in learning or cognitive strategies to perform a given task) could explain differences between subjects in the speed and amount of aphasia recovery (Seghier & Price, 2018). As Seghier and Price (2018) suggest, we could derive the likelihood of recovery of PWA from the amount of variability in the functional response in a normal population. In a healthy population, there are multiple ways to perform a certain (language) task, and therefore, PWA should be able to compensate for a problem in that specific language task. Seghier and Price (2018) propose the method of covariance analysis to characterize interindividual variability, which typically is masked in group analyses. However, once again, this requires a large number of observations from a large number of individuals and thus a large study sample size.

In addition, measurement and characterization of interindividual differences at the neural level in a heterogeneous group of PWA would enable the tailoring of appropriate interventions to every individual. For example, in the context of transcranial direct-current stimulation, Shah-Basak et al. (2015) demonstrated that patients with more extensive lesions in the frontal lobe benefited more from left-hemispheric inhibition, while PWA with less frontal damage responded better to left-hemispheric facilitation. On the other hand, in large databases of stroke patients with and without aphasia such as the Predicting Language Outcome and Recovery after Stroke (PLORAS)-database (Seghier et al., 2016), machine learning approaches could be used to estimate the response to intervention in PWA. By comparing neuroimaging data of a new PWA with neuroimaging data of the stroke database and the treatment outcomes, the system could learn which treatment is generally effective for PWA with similar neuroimaging characteristics. This could be done with lesion data, as well as with structural and functional connectivity patterns (Silasi & Murphy, 2014).

### Conclusion

Across treatments and participants, the regions that are involved in language recovery are very diverse. Similarity between intervention studies targeting the same linguistic component is not apparently greater than similarity between intervention studies targeting different linguistic components. However, methodological shortcomings and variability between studies make it hard to present clear conclusions on treatment-related brain changes in PWA. It is possible that treatment-related brain changes associated with recovery of language after brain damage entail both regions traditionally involved in linguistic processing as well as regions involved in other cognitive functions in both hemispheres. If this is true, we should interpret recovery from aphasia as the result of the adaptive reorganization of functionally heterogeneous perilesional and bilateral neural networks not uniquely involved in language processing (Cahana-Amitay & Albert, 2015). Therefore, we argue for the interpretation of treatment-related language recovery in light of the concept of *neural multifunctionality* (as discussed in the review of Cahana-Amitay & Albert, 2015). This label highlights the constant and dynamic interactions between neural networks supporting linguistic as well as non-linguistic functions, such as cognitive, emotional, and sensorimotor processing. In other words, the language network is most likely widely distributed over many different functionally and structurally connected brain regions that are activated interactively. Specific linguistic functions are fulfilled through the integration of neural activity in many regions subserving many functions (Price, 2012).

## ACKNOWLEDGMENTS

The Fonds Wetenschappelijk Onderzoek, Flanders, Belgium, provided funding for this project, including a grant from the Strategic Basic Research Programme.

## FUNDING INFORMATION

Klara Schevenels, Fonds Wetenschappelijk Onderzoek (http://dx.doi.org/10.13039/501100003130), Award ID: 1S81620N. Maaike Vandermosten, Fonds Wetenschappenlijk Onderzoek (http://dx.doi.org/10.13039/501100003130), Award ID: 1521819N.

## AUTHOR CONTRIBUTIONS

Klara Schevenels and Maaike Vandermosten conceived and planned the review. Klara Schevenels took the lead in writing the manuscript. All authors provided critical feedback and helped shape the review.

## References

[bib1] Abel, S. , Weiller, C. , Huber, W. , & Willmes, K. (2014). Neural underpinnings for model-oriented therapy of aphasic word production. Neuropsychologia, 57(1), 154–165. 10.1016/j.neuropsychologia.2014.03.010 24686092

[bib2] Abel, S. , Weiller, C. , Huber, W. , Willmes, K. , & Specht, K. (2015). Therapy-induced brain reorganization patterns in aphasia. Brain, 138(4), 1097–1112. 10.1093/brain/awv022 25688082

[bib3] Ackermann, H. , & Riecker, A. (2010). The contribution(s) of the insula to speech production: A review of the clinical and functional imaging literature. Brain Structure and Function, 214(5–6), 419–433. 10.1007/s00429-010-0257-x 20512374

[bib4] Aerts, A. , Batens, K. , Santens, P. , van Mierlo, P. , Hartsuiker, R. , Hemelsoet, D. , … De Letter, M. (2015). Aphasia therapy early after stroke: Behavioural and neurophysiological changes in the acute and post-acute phases. Aphasiology, 29(7), 845–871. 10.1080/02687038.2014.996520

[bib5] Baum, S. R. , & Pell, M. D. (1999). The neural bases of prosody: Insights from lesion studies and neuroimaging. Aphasiology, 13(8), 581–608. 10.1080/026870399401957

[bib6] Benjamin, M. L. , Towler, S. , Garcia, A. , Park, H. , Sudhyadhom, A. , Harnish, S. , … Crosson, B. (2014). A behavioral manipulation engages right frontal cortex during aphasia therapy. Neurorehabilitation and Neural Repair, 28(6), 545–553. 10.1177/1545968313517754 24407914 PMC4090303

[bib7] Bonakdarpour, B. , Beeson, P. M. , Demarco, A. T. , & Rapcsak, S. Z. (2015). Variability in blood oxygen level dependent (BOLD) signal in patients with stroke-induced and primary progressive aphasia. NeuroImage: Clinical, 8, 87–94. 10.1016/j.nicl.2015.03.014 26106531 PMC4473284

[bib8] Bonakpardour, B. , Parrish, T. B. , & Thompson, C. K. (2007). Hemodynamic response function in patients with stroke-induced aphasia: Implications for fMRI data analysis. NeuroImage, 36(2), 322–331. https://www.sciencedirect.com/science/article/abs/pii/S1053811907001371?via%3Dihub 17467297 10.1016/j.neuroimage.2007.02.035 PMC2041913

[bib9] Bonilha, L. , Gleichgerrcht, E. , Nesland, T. , Rorden, C. , & Fridriksson, J. (2016). Success of anomia treatment in aphasia is associated with preserved architecture of global and left temporal lobe structural networks. Neurorehabilitation and Neural Repair, 30(3), 266–279. https://journals.sagepub.com/doi/10.1177/1545968315593808?url_ver=Z39.88-2003&rfr_id=ori:rid:crossref.org&rfr_dat=cr_pub%3dpubmed 26150147 10.1177/1545968315593808 PMC4703576

[bib10] Bonilha, L. , Hillis, A. E. , Hickok, G. , den Ouden, D. B. , Rorden, C. , & Fridriksson, J. (2017). Temporal lobe networks supporting the comprehension of spoken words. Brain, 140(9), 2370–2380. 10.1093/brain/awx169 29050387 PMC8189019

[bib11] Boyke, J. , Driemeyer, J. , Gaser, C. , Buchel, C. , & May, A. (2008). Training-induced brain structure changes in the elderly. Journal of Neuroscience, 28(28), 7031–7035. 10.1523/JNEUROSCI.0742-08.2008 18614670 PMC6670504

[bib12] Brady, M. , Kelly, H. , Godwin, J. , Enderby, P. , & Campbell, P. (2016). Speech and language therapy for aphasia following stroke. Cochrane Database of Systematic Reviews, 6, 4–7. 10.1002/14651858.CD000425.pub4 PMC8078645 27245310

[bib13] Breier, J. I. , Juranek, J. , & Papanicolaou, A. C. (2011). Changes in maps of language function and the integrity of the arcuate fasciculus after therapy for chronic aphasia. Neurocase, 17(6), 506–517. 10.1080/13554794.2010.547505 22111962 PMC3278272

[bib14] Brownsett, S. L. E. , Warren, J. E. , Geranmayeh, F. , Woodhead, Z. , Leech, R. , & Wise, R. J. S. (2014). Cognitive control and its impact on recovery from aphasic stroke. Brain, 137(1), 242–254. 10.1093/brain/awt289 24163248 PMC3891442

[bib15] Cabeza, R. , Anderson, N. D. , Locantore, J. K. , & McIntosh, A. R. (2002). Aging gracefully: Compensatory brain activity in high-performing older adults. NeuroImage, 17(3), 1394–1402. 10.1006/nimg.2002.1280 12414279

[bib16] Cahana-Amitay, D. , & Albert, M. L. (2015). Neuroscience of aphasia recovery: The concept of neural multifunctionality. Current Neurology and Neuroscience Reports, 15(7). 10.1007/s11910-015-0568-7 26008816

[bib17] Carter, A. R. , Shulman, G. L. , & Corbetta, M. (2012). Why use a connectivity-based approach to study stroke and recovery of function? NeuroImage, 62(4), 2271–2280. 10.1016/j.neuroimage.2012.02.070 22414990 PMC3733251

[bib18] Catani, M. , Jones, D. K. , & Ffytche, D. H. (2005). Perisylvian language networks of the human brain. Annals of Neurology, 57(1), 8–16. 10.1002/ana.20319 15597383

[bib19] Cavanna, A. E. , & Trimble, M. R. (2006). The precuneus: A review of its functional anatomy and behavioural correlates. Brain, 129(3), 564–583. 10.1093/brain/awl004 16399806

[bib20] Chein, J. M. , & Schneider, W. (2005). Neuroimaging studies of practice-related change: fMRI and meta-analytic evidence of a domain-general control network for learning. Cognitive Brain Research, 25(3), 607–623. 10.1016/j.cogbrainres.2005.08.013 16242923

[bib21] Cocquyt, E.-M. , De Ley, L. , Santens, P. , Van Borsel, J. , & De Letter, M. (2017). The role of the right hemisphere in the recovery of stroke-related aphasia: A systematic review. Journal of Neurolinguistics, 44, 68–90. 10.1016/j.jneuroling.2017.03.004

[bib22] Cornelissen, K. , Laine, M. , Tarkiainen, A. , Järvensivu, T. , Martin, N. , & Salmelin, R. (2003). Adult brain plasticity elicited by anomia treatment. Journal of Cognitive Neuroscience, 15(3), 444–461. 10.1162/089892903321593153 12729495

[bib23] Crinion, J. , Holland, A. L. , Copland, D. A. , Thompson, C. K. , & Hillis, A. E. (2013). Neuroimaging in aphasia treatment research: Quantifying brain lesions after stroke. NeuroImage, 73, 208–214. 10.1016/j.neuroimage.2012.07.044 22846659 PMC3534842

[bib24] Crinion, J. T. , & Leff, A. P. (2007). Recovery and treatment of aphasia after stroke: Functional imaging studies. Current Opinion in Neurology, 20, 667–673. 17992087 10.1097/WCO.0b013e3282f1c6fa

[bib25] Crosson, B. , McGregor, K. , Gopinath, K. S. , Conway, T. W. , Benjamin, M. , Chang, Y. L. , … White, K. D. (2007). Functional MRI of language in aphasia: A review of the literature and the methodological challenges. Neuropsychology Review, 17(2), 157–177. 10.1007/s11065-007-9024-z 17525865 PMC2659355

[bib26] Dahlberg, C. , Hawley, L. , Morey, C. , Newman, J. , Cusick, C. P. , & Harrison-Felix, C. (2006). Social communication skills in persons with post-acute traumatic brain injury: Three perspectives. Brain Injury, 20(4), 425–435. 10.1080/02699050600664574 16716988

[bib27] Eickhoff, S. B. , Nichols, T. E. , Laird, A. R. , Hoffstaedter, F. , Amunts, K. , Fox, P. T. , … Eickhoff, C. R. (2016). Behavior, sensitivity, and power of activation likelihood estimation characterized by massive empirical simulation. NeuroImage, 137, 70–85. 10.1016/j.neuroimage.2016.04.072 27179606 PMC4981641

[bib28] Forkel, S. J. , de Schotten, M. T. , Dell’Acqua, F. , Kalra, L. , Murphy, D. G. M. , Williams, S. C. R. , & Catani, M. (2014). Anatomical predictors of aphasia recovery: A tractography study of bilateral perisylvian language networks. Brain, 137(7), 2027–2039. 10.1093/brain/awu113 24951631

[bib29] Fridriksson, J. (2010). Preservation and modulation of specific left hemisphere regions is vital for treated recovery from anomia in stroke. Journal of Neurophysiology, 30(35), 11558–11564. 10.1523/JNEUROSCI.2227-10.2010 PMC2938788 20810877

[bib30] Fridriksson, J. , Hubbard, H. I. , Hudspeth, S. G. , Holland, A. L. , Bonilha, L. , Fromm, D. , & Rorden, C. (2012). Speech entrainment enables patients with Broca’s aphasia to produce fluent speech. Brain, 135(12), 3815–3829. 10.1093/brain/aws301 23250889 PMC3525061

[bib31] Fridriksson, J. , & Morrow, L. (2005). Cortical activation and language task difficulty in aphasia. Aphasiology, 19(3–5), 239–250. 10.1080/02687030444000714 16823468 PMC1486765

[bib32] Fridriksson, J. , Morrow-Odom, L. , Moser, D. , Fridriksson, A. , & Baylis, G. (2006). Neural recruitment associated with anomia treatment in aphasia. NeuroImage, 32(3), 1403–1412. 10.1016/j.neuroimage.2006.04.194 16766207

[bib33] Fridriksson, J. , Moser, D. , Bonilha, L. , Morrow-Odom, K. L. , Shaw, H. , Fridriksson, A. , Baylis, G. C. , & Rorden, C. (2007). Neural correlates of phonological and semantic-based anomia treatment in aphasia. Neuropsychologia, 45(8), 1812–1822. 10.1016/j.neuropsychologia.2006.12.017 17292928 PMC2547988

[bib34] Fridriksson, J. , Richardson, J. D. , Fillmore, P. , & Cai, B. (2012). Left hemisphere plasticity and aphasia recovery. NeuroImage, 60(2), 854–863. 10.1016/j.neuroimage.2011.12.057 22227052 PMC3313653

[bib35] Friederici, A. D. (2011). The brain basis of language processing: From structure to function. Physiological Reviews, 91(4), 1357–1392. 10.1152/physrev.00006.2011 22013214

[bib36] Geranmayeh, F. , Brownsett, S. L. E. , & Wise, R. J. S. (2014). Task-induced brain activity in aphasic stroke patients: What is driving recovery? Brain, 137(10), 2632–2648. 10.1093/brain/awu163 24974382 PMC4163030

[bib37] Geschwind, N. (1965). Disconnexion syndromes in animal and man: Part II. Brain, 88, 585–644. 10.1093/brain/88.3.585 5318824

[bib38] Geschwind, N. (1970). The organization of language and the brain. Science, 170(3961), 940–944. 10.1126/science.170.3961.940 5475022

[bib39] Geschwind, N. (2010). Disconnexion syndromes in animal and man: Part I. Neuropsychology Review, 20, 127–157. Original work published 1965. 10.1007/978-94-010-2093-0_8 20540177

[bib40] Gili, T. , Fiori, V. , De Pasquale, G. , Sabatini, U. , Caltagirone, C. , & Marangolo, P. (2017). Right sensory-motor functional networks subserve action observation therapy in aphasia. Brain Imaging and Behavior, 11(5), 1397–1411. 10.1007/s11682-016-9635-1 27734301

[bib41] Gold, B. T. , & Kertesz, A. (2000). Right hemisphere semantic processing of visual words in an aphasic patient: An fMRI study. Brain and Language, 73(3), 456–465. 10.1006/brln.2000.2317 10860566

[bib42] Griffis, J. C. , Nenert, R. , Allendorfer, J. B. , & Szaflarski, J. P. (2017). Damage to white matter bottlenecks contributes to language impairments after left hemispheric stroke. NeuroImage: Clinical, 14, 552–565. 10.1016/j.nicl.2017.02.019 28337410 PMC5350568

[bib43] Hagoort, P. (2014). Nodes and networks in the neural architecture for language: Broca’s region and beyond. Current Opinion in Neurobiology, 28, 136–141. 10.1016/j.conb.2014.07.013 25062474

[bib44] Haldin, C. , Acher, A. , Kauffmann, L. , Hueber, T. , Cousin, E. , Badin, P. , … Baciu, M. (2018). Speech recovery and language plasticity can be facilitated by Sensori-Motor Fusion training in chronic non-fluent aphasia. A case report study. Clinical Linguistics and Phonetics, 32(7), 595–621. 10.1080/02699206.2017.1402090 29148845

[bib45] Hartwigsen, G. , & Saur, D. (2019). Neuroimaging of stroke recovery from aphasia –Insights into plasticity of the human language network. NeuroImage, 190, 14–31. 10.1016/j.neuroimage.2017.11.056 29175498

[bib46] Heiss, W. , & Thiel, A. (2006). A proposed regional hierarchy in recovery of post-stroke aphasia. Brain and Language, 98, 118–123. 10.1016/j.bandl.2006.02.002 16564566

[bib47] Hickok, G. , & Poeppel, D. (2004). Dorsal and ventral streams: A framework for understanding aspects of the functional anatomy of language. Cognition, 92(1–2), 67–99. 10.1016/j.cognition.2003.10.011 15037127

[bib48] Hickok, G. , & Poeppel, D. (2007). The cortical organization of speech processing. Nature Reviews Neurology, 8, 393–402. 10.1038/nrn2113 17431404

[bib49] Honey, C. J. , Sporns, O. , Cammoun, L. , Gigandet, X. , Thiran, J. P. , Meuli, R. , & Hagmann, P. (2009). Predicting human resting-state functional connectivity from structural connectivity. Proceedings of the National Academy of Sciences, 106(6), 2035–2040. 10.1073/pnas.0811168106 PMC2634800 19188601

[bib50] Hosomi, A. , Nagakane, Y. , & Yamada, K. (2009). Assessment of arcuate fasciculus with diffusion-tensor tractography may predict the prognosis of aphasia in patients with left middle cerebral artery infarcts. Diagnostic Neuroradiology, 51, 549–555. 10.1007/s00234-009-0534-7 19434402

[bib51] Humphreys, G. F. , & Lambon Ralph, M. A. (2015). Fusion and fission of cognitive functions in the human parietal cortex. Cerebral Cortex, 25(10), 3547–3560. 10.1093/cercor/bhu198 25205661 PMC4585503

[bib52] Jang, S. H. (2013). Diffusion tensor imaging studies on arcuate fasciculus in stroke patients: A review. Frontiers in Human Neuroscience, 7(November), 1–7. 10.3389/fnhum.2013.00749 24198780 PMC3814569

[bib53] Jang, S. H. , & Lee, H. D. (2014). Recovery of injured arcuate fasciculus in the dominant hemisphere in a patient with an intracerebral hemorrhage. American Journal of Physical Medicine and Rehabilitation, 93(12), e15–e18. 10.1097/PHM.0000000000000202 25299531

[bib54] Jungblut, M. , Huber, W. , Mais, C. , & Schnitker, R. (2014). Paving the way for speech: Voice-training-induced plasticity in chronic aphasia and apraxia of apeech—three single cases. Neural Plasticity, 2014, 1–14. 10.1155/2014/841982 PMC4058170 24977055

[bib55] Kaye, R. C. , & Cherney, L. R. (2016). Script templates: A practical approach to script training in aphasia. Topics in Language Disorders, 36(2), 136–153. 10.1097/TLD.0000000000000086 27594730 PMC5006751

[bib56] Kiran, S. , Meier, E. L. , Kapse, K. J. , & Glynn, P. A. (2015). Changes in task-based effective connectivity in language networks following rehabilitation in post-stroke patients with aphasia. Frontiers in Human Neuroscience, 9(June), 1–20. 10.3389/fnhum.2015.00316 26106314 PMC4460429

[bib57] Kurland, J. , Baldwin, K. , & Tauer, C. (2010). Treatment-induced neuroplasticity following intensive naming therapy in a case of chronic Wernicke’s aphasia. Aphasiology, 24(6–8), 737–751. 10.1080/02687030903524711

[bib58] Lake, E. M. R. , Bazzigaluppi, P. , & Stefanovic, B. (2016). Functional magnetic resonance imaging in chronic ischaemic stroke. Philosophical Transactions of the Royal Society B: Biological Sciences, 371(1705), 1–11. 10.1098/rstb.2015.0353 PMC5003855 27574307

[bib59] Leon, S. A. , Rodriguez, A. D. , & Rosenbek, J. C. (2015). Right hemisphere damage and prosody. In A. M. Raymer & L. Gonzalez-Rothi (Eds.), The Oxford Handbook of Aphasia and Language Disorders, Oxford Handbooks Online. 10.1093/oxfordhb/9780199772391.013.15

[bib60] Leonard, C. , Laird, L. , Burianová, H. , Graham, S. , Grady, C. , Simic, T. , & Rochon, E. (2015). Behavioural and neural changes after a “choice” therapy for naming deficits in aphasia: Preliminary findings. Aphasiology, 29(4), 506–525. 10.1080/02687038.2014.971099

[bib61] Madan, C. R. (2015). Creating 3D visualizations of MRI data: A brief guide. F1000Research, 466, 1–13. 10.12688/f1000research.6838.1 PMC4648228 26594340

[bib62] Marchina, S. , Zhu, L. L. , Norton, A. , Zipse, L. , Wan, C. Y. , & Schlaug, G. (2011). Impairment of speech production predicted by lesion load of the left arcuate fasciculus. Stroke, 42, 2251–2256. 10.1161/STROKEAHA.110.606103 21719773 PMC3167233

[bib63] Marcotte, K. , Adrover-Roig, D. , Damien, B. , de Préaumont, M. , Généreux, S. , Hubert, M. , & Ansaldo, A. I. (2012). Therapy-induced neuroplasticity in chronic aphasia. Neuropsychologia, 50(8), 1776–1786. 10.1016/j.neuropsychologia.2012.04.001 22564481

[bib64] Marcotte, K. , & Ansaldo, A. I. (2010). The neural correlates of semantic feature analysis in chronic aphasia: Discordant patterns according to the etiology. Seminars in Speech and Language, 31(1), 52–63. 10.1055/s-0029-1244953 20221954

[bib65] Marcotte, K. , Laird, L. , Bitan, T. , Meltzer, J. A. , Graham, S. J. , Leonard, C. , & Rochon, E. (2018). Therapy-induced neuroplasticity in chronic aphasia after phonological component analysis: A matter of intensity. Frontiers in Neurology, 9(APR), 1–7. 10.3389/fneur.2018.00225 29686646 PMC5900891

[bib66] Marcotte, K. , Perlbarg, V. , Marrelec, G. , Benali, H. , & Ansaldo, A. I. (2013). Default-mode network functional connectivity in aphasia: Therapy-induced neuroplasticity. Brain and Language, 124(1), 45–55. 10.1016/j.bandl.2012.11.004 23274798

[bib67] McKinnon, E. T. , Fridriksson, J. , Glenn, G. R. , Jensen, J. H. , Helpern, J. A. , Basilakos, A. , … Bonilha, L. (2017). Structural plasticity of the ventral stream and aphasia recovery. Annals of Neurology, 82(1), 147–151. 10.1002/ana.24983 28628946 PMC5559663

[bib68] Meinzer, M. , Beeson, P. M. , Cappa, S. , Crinion, J. , Kiran, S. , Saur, D. , … Thompson, C. K. (2013). Neuroimaging in aphasia treatment research: Consensus and practical guidelines for data analysis. NeuroImage, 73, 215–224. 10.1016/j.neuroimage.2012.02.058 22387474 PMC3416913

[bib69] Menenti, L. , Gierhan, S. M. E. , Segaert, K. , & Hagoort, P. (2011). Shared language: Overlap and segregation of the neuronal infrastructure for speaking and listening revealed by functional MRI. Psychological Science, 22(9), 1173–1182. 10.1177/0956797611418347 21841148

[bib70] Menke, R. , Meinzer, M. , Kugel, H. , Deppe, M. , Baumgärtner, A. , Schiffbauer, H. , … Breitenstein, C. (2009). Imaging short- and long-term training success in chronic aphasia. BMC Neuroscience, 10, 118. 10.1186/1471-2202-10-118 19772660 PMC2754483

[bib71] Meyer, M. N. (2018). Practical tips for ethical data sharing. Advances in Methods and Practices in Psychological Science, 1(1), 131–144. 10.1177/2515245917747656 PMC6544443 31157320

[bib72] Murphy, T. H. , & Corbett, D. (2009). Plasticity during stroke recovery: From synapse to behaviour. Nature Reviews Neuroscience, 10(12), 861–872. 10.1038/nrn2735 19888284

[bib73] Nair, V. A. , Raut, R. V. , & Prabhakaran, V. (2017). Investigating the blood oxygenation level-dependent functional MRI response to a verbal fluency task in early stroke before and after hemodynamic scaling. Frontiers in Neurology, 8(JUN). 10.3389/fneur.2017.00283 PMC5474460 28674515

[bib74] Nardo, D. , Holland, R. , Leff, A. P. , Price, C. J. , & Crinion, J. T. (2017). Less is more: Neural mechanisms underlying anomia treatment in chronic aphasic patients. Brain, 140(11), 3039–3054. 10.1093/brain/awx234 29053773 PMC5808641

[bib75] NIDCD. (2015). Fact sheet: Aphasia. NIH Pub. No. 97-4257. PDF Retrieved from https://www.nidcd.nih.gov/sites/default/files/Documents/health/voice/Aphasia.pdf

[bib76] Ogar, J. , Slama, H. , Dronkers, N. , Amici, S. , & Gorno-Tempini, M. L. (2005). Apraxia of speech: An overview. Neurocase, 11(6), 427–432. 10.1080/13554790500263529 16393756

[bib77] Plowman, E. , Hentz, B. , & Ellis, C. (2012). Post-stroke aphasia prognosis: A review of patient-related and stroke-related factors. Journal of Evaluation in Clinical Practice, 18, 689–694. 10.1111/j.1365-2753.2011.01650.x 21395923

[bib78] Poeppel, D. (2003). The analysis of speech in different temporal integration windows: Cerebral lateralization as “asymmetric sampling in time.” Speech Communication, 41(1), 245–255. 10.1016/S0167-6393(02)00107-3

[bib79] Poldrack, R. A. , & Gorgolewski, K. J. (2014). Making big data open: Data sharing in neuroimaging. Nature Neuroscience, 17(11), 1510–1517. 10.1038/nn.3818 25349916

[bib80] Poldrack, R. A. , Mumford, J. A. , & Nichols, T. E. (2011). Handbook of functional MRI data analysis. Cambridge, UK: Cambridge University Press.

[bib81] Price, C. J. (2000). The anatomy of language: Contributions from functional neuroimaging. Journal of Anatomy, 197(3), 335–359. 10.1046/j.1469-7580.2000.19730335.x 11117622 PMC1468137

[bib82] Price, C. J. (2010). The anatomy of language: A review of 100 fMRI studies published in 2009. Annals of the New York Academy of Sciences, 1191, 62–88. 10.1111/j.1749-6632.2010.05444.x 20392276

[bib83] Price, C. J. (2012). A review and synthesis of the first 20 years of PET and fMRI studies of heard speech, spoken language and reading. NeuroImage, 62(2), 816–847. 10.1016/j.neuroimage.2012.04.062 22584224 PMC3398395

[bib84] Price, C. J. , & Crinion, J. (2005). The latest on functional imaging studies of aphasic stroke. Current Opinion in Neurology, 18(4), 429–434. 10.1097/01.wco.0000168081.76859.c1 16003120

[bib85] Price, C. J. , Crinion, J. , & Friston, K. J. (2006). Design and analysis of fMRI studies with neurologically impaired patients. Journal of Magnetic Resonance Imaging, 23, 816–826. 10.1002/jmri.20580 16649208

[bib86] Price, C. J. , Seghier, M. L. , & Leff, A. P. (2010). Predicting language outcome and recovery after stroke: The PLORAS system. Nature Reviews Neurology, 6(4), 202–210. 10.1038/nrneurol.2010.15 20212513 PMC3556582

[bib87] Pulvermüller, F. , & Berthier, M. L. (2008). Aphasia therapy on a neuroscience basis. Aphasiology, 22(6), 563–599. 10.1080/02687030701612213 18923644 PMC2557073

[bib88] Pulvermüller, F. , Neininger, B. , Elbert, T. , Mohr, B. , Rockstroh, B. , Koebbel, P. , & Taub, E. (2001). Constraint-induced therapy of chronic aphasia after stroke. Stroke, 32, 2–7. 10.1161/01.STR.32.7.1621 11441210

[bib89] Raboyeau, G. , De Boissezon, X. , Marie, N. , Balduyck, S. , Puel, M. , Bézy, C. , … Cardebat, D. (2008). Right hemisphere activation in recovery from aphasia: Lesion effect or function recruitment? Neurology, 70, 290–298. 10.1212/01.wnl.0000287115.85956.87 18209203

[bib90] Raichle, M. E. , Fiez, J. A. , Videen, T. O. , MacLeod, A.-M. K. , Pardo, J. V. , Fox, P. T. , & Petersen, S. E. (1994). Practice-related changes in human brain functional anatomy during nonmotor learning. Cerebral Cortex, 4(1), 8–26. 10.1093/cercor/4.1.8 8180494

[bib91] Ramus, F. , Altarelli, I. , Jednoróg, K. , Zhao, J. , & Scotto di Covella, L. (2018). Neuroanatomy of developmental dyslexia: Pitfalls and promise. Neuroscience and Biobehavioral Reviews, 84, 434–452. 10.1016/j.neubiorev.2017.08.001 28797557

[bib92] Rapp, B. , Caplan, D. , Edwards, S. , Visch-Brink, E. , & Thompson, C. K. (2013). Neuroimaging in aphasia treatment research: Issues of experimental design for relating cognitive to neural changes. NeuroImage, 73, 200–207. 10.1016/j.neuroimage.2012.09.007 22974976 PMC3600065

[bib93] Rasmussen, T. , & Milner, B. (1977). The role of early left-brain injury in determining lateralization of cerebral speech functions. Annals of the New York Academy of Sciences, 299(1), 355–369. 10.1111/j.1749-6632.1977.tb41921.x 101116

[bib94] Rochon, E. , Leonard, C. , Burianova, H. , Laird, L. , Soros, P. , Graham, S. , & Grady, C. (2010). Neural changes after phonological treatment for anomia: An fMRI study. Brain and Language, 114(3), 164–179. 10.1016/j.bandl.2010.05.005 20547416 PMC4898952

[bib95] Sandberg, C. W. , Bohland, J. W. , & Kiran, S. (2015). Changes in functional connectivity related to direct training and generalization effects of a word finding treatment in chronic aphasia. Brain and Language, 150, 103–116. 10.1016/j.bandl.2015.09.002 26398158 PMC4663144

[bib96] Santhanam, P. , Duncan, E. S. , & Small, S. L. (2018). Therapy-induced plasticity in chronic aphasia is associated with behavioral improvement and time since stroke. Brain Connectivity, 8(3), 179–188. 10.1089/brain.2017.0508 29338310 PMC5899281

[bib97] Saur, D. , & Hartwigsen, G . (2012). Neurobiology of language recovery after stroke: Lessons from neuroimaging studies. Archives of Physical Medicine and Rehabilitation, 93(1), S15–S25. 10.1016/j.apmr.2011.03.036 22202187

[bib98] Saur, D. , Kreher, B. W. , Schnell, S. , Kummerer, D. , Kellmeyer, P. , Vry, M.-S. , … Weiller, C. (2008). Ventral and dorsal pathways for language. Proceedings of the National Academy of Sciences, 105(46), 18035–18040. 10.1073/pnas.0805234105 PMC2584675 19004769

[bib99] Saur, D. , Lange, R. , Baumgaertner, A. , Schraknepper, V. , Willmes, K. , Rijntjes, M. , & Weiller, C. (2006). Dynamics of language reorganization after stroke. Brain, 129(6), 1371–1384. 10.1093/brain/awl090 16638796

[bib100] Schlaug, G. , Marchina, S. , & Norton, A. (2008). From singing to speaking: Why singing may lead to recovery of expressive language function in patients with Broca’s aphasia. Music Perception, 25(4), 315–323. 10.1525/MP.2008.25.4.315 21197418 PMC3010734

[bib101] Schlaug, G. , Marchina, S. , & Norton, A. (2009). Evidence for plasticity in white-matter tracts of patients with chronic Broca’s aphasia undergoing intense intonation-based speech therapy. Annals of the New York Academy of Sciences, 1169, 385–394. 10.1111/j.1749-6632.2009.04587.x 19673813 PMC2777670

[bib102] Scholz, J. , Klein, M. C. , Behrens, T. E. J. , & Johansen-Berg, H. (2009). Training induces changes in white-matter architecture. Nature Neuroscience, 12(11), 1370–1371. 10.1038/nn.2412 19820707 PMC2770457

[bib103] Segaert, K. , Menenti, L. , Weber, K. , Petersson, K. M. , & Hagoort, P. (2012). Shared syntax in language production and language comprehension —An fMRI study. Cerebral Cortex, 22(7), 1662–1670. 10.1093/cercor/bhr249 21934094 PMC3377967

[bib104] Seghier, M. L. , Patel, E. , Prejawa, S. , Ramsden, S. , Selmer, A. , Lim, L. , … Price, C. J. (2016). The PLORAS database: A data repository for predicting language outcome and recovery after stroke. NeuroImage, 124, 1208–1212. 10.1016/j.neuroimage.2015.03.083 25882753 PMC4658335

[bib105] Seghier, M. L. , & Price, C. J. (2018). Interpreting and utilising intersubject variability in brain function. Trends in Cognitive Sciences, 22(6), 517–530. 10.1016/j.tics.2018.03.003 29609894 PMC5962820

[bib106] Shah-Basak, P. P. , Norise, C. , Garcia, G. , Torres, J. , Faseyitan, O. , & Hamilton, R. H. (2015). Individualized treatment with transcranial direct current stimulation in patients with chronic non-fluent aphasia due to stroke. Frontiers in Human Neuroscience, 9(201), 1–12. 10.3389/fnhum.2015.00201 25954178 PMC4404833

[bib107] Siegel, J. S. , Ramsey, L. E. , Snyder, A. Z. , Metcalf, N. V. , Chacko, R. V. , Weinberger, K. , … Corbetta, M. (2016). Disruptions of network connectivity predict impairment in multiple behavioral domains after stroke. Proceedings of the National Academy of Sciences, 113(30), E4367–E4376. 10.1073/pnas.1521083113 PMC4968743 27402738

[bib108] Siegel, J. S. , Shulman, G. L. , & Corbetta, M. (2017). Measuring functional connectivity in stroke: Approaches and considerations. Journal of Cerebral Blood Flow and Metabolism, 37(8), 2665–2678. 10.1177/0271678X17709198 28541130 PMC5536814

[bib109] Siegel, J. S. , Snyder, A. Z. , Ramsey, L. , Shulman, G. L. , & Corbetta, M. (2016). The effects of hemodynamic lag on functional connectivity and behavior after stroke. Journal of Cerebral Blood Flow and Metabolism, 36(12), 2162–2176. 10.1177/0271678X15614846 26661223 PMC5363662

[bib110] Silasi, G. , & Murphy, T. H. (2014). Stroke and the connectome: How connectivity guides therapeutic intervention. Neuron, 83(6), 1354–1368. 10.1016/j.neuron.2014.08.052 25233317

[bib111] Stegemöller, E. L. (2017). The neuroscience of speech and language. Music Therapy Perspectives, 35(2), 107–112. 10.1093/mtp/mix007

[bib112] Stevens, M. C. , Kiehl, K. A. , Pearlson, G. D. , & Calhoun, V. D. (2009). Brain network dynamics during error commission. Human Brain Mapping, 30(1), 24–37. 10.1002/hbm.20478 17979124 PMC2669663

[bib113] Stokes, R. C. , Venezia, J. H. , & Hickok, G. (2019). The motor system’s [modest] contribution to speech perception. Psychonomic Bulletin & Review, 26, 1354–1366. 10.3758/s13423-019-01580-2 30945170 PMC9539476

[bib114] Tabei, K. I. , Satoh, M. , Nakano, C. , Ito, A. , Shimoji, Y. , Kida, H. , … Tomimoto, H. (2016). Improved neural processing efficiency in a chronic aphasia patient following melodic intonation therapy: A neuropsychological and functional MRI study. Frontiers in Neurology, 7(SEP), 1–6. 10.3389/fneur.2016.00148 27698650 PMC5027199

[bib115] Thompson, C. K. , den Ouden, D. B. , Bonakdarpour, B. , Garibaldi, K. , & Parrish, T. B. (2010). Neural plasticity and treatment-induced recovery of sentence processing in agrammatism. Neuropsychologia, 48(11), 3211–3227. https://www.sciencedirect.com/science/article/abs/pii/S0028393210002757?via%3Dihub 20603138 10.1016/j.neuropsychologia.2010.06.036 PMC3164559

[bib116] Thompson, C. K. , Riley, E. A. , den Ouden, D. B. , Meltzer-Asscher, A. , & Lukic, S. (2013). Training verb argument structure production in agrammatic aphasia: Behavioral and neural recovery patterns. Cortex, 49(9), 2358–2376. 10.1016/j.cortex.2013.02.003 23514929 PMC3759546

[bib117] Tourville, J. A. , & Guenther, F. H. (2011). The DIVA model: A neural theory of speech acquisition and production. Language and Cognitive Processes, 26(7), 1–27. https://www.tandfonline.com/doi/abs/10.1080/01690960903498424 10.1080/01690960903498424 PMC3650855 23667281

[bib118] Tremblay, P. , & Dick, A. S. (2016). Broca and Wernicke are dead, or moving past the classic model of language neurobiology. Brain and Language, 162, 60–71. 10.1016/j.bandl.2016.08.004 27584714

[bib119] Tsumoto, T. (1992). Long-term potentiation and long-term depression in the neocortex. Progress in Neurobiology, 39(2), 209–228. 10.1016/0301-0082(92)90011-3 1323860

[bib120] Turkeltaub, P. E. , Messing, S. , Norise, C. , & Hamilton, R. H. (2011). Are networks for residual language function and recovery consistent across aphasic patients? Neurology, 76(20), 1726–1734. 10.1212/WNL.0b013e31821a44c1 21576689 PMC3100133

[bib121] Tzourio-Mazoyer, N. , Landeau, B. , Papathanassiou, D. , Crivello, F. , Etard, O. , Delcroix, N. , … Joliot, M. (2002). Automated anatomical labeling of activations in SPM using a macroscopic anatomical parcellation of the MNI MRI single-subject brain. NeuroImage, 15(1), 273–289. 10.1006/nimg.2001.0978 11771995

[bib122] van Hees, S. , McMahon, K. , Angwin, A. , de Zubicaray, G. , & Copland, D. A. (2014). Neural activity associated with semantic versus phonological anomia treatments in aphasia. Brain and Language, 129(1), 47–57. 10.1016/j.bandl.2013.12.004 24556337

[bib123] van Hees, S. , McMahon, K. , Angwin, A. , de Zubicaray, G. , Read, S. , & Copland, D. A. (2014a). Changes in white matter connectivity following therapy for anomia post stroke. Neurorehabilitation and Neural Repair, 28(4), 325–334. 10.1177/1545968313508654 24297762

[bib124] van Hees, S. , McMahon, K. , Angwin, A. , de Zubicaray, G. , Read, S. , & Copland, D. A. (2014b). A functional MRI study of the relationship between naming treatment outcomes and resting state functional connectivity in post-stroke aphasia. Human Brain Mapping, 35(8), 3919–3931. 10.1002/hbm.22448 24453137 PMC6869730

[bib125] Varley, R. (2011). Rethinking aphasia therapy: A neuroscience perspective. International Journal of Speech-Language Pathology, 13(1), 11–20. 10.3109/17549507.2010.497561 21329406

[bib126] Vigneau, M. , Beaucousin, V. , Hervé, P. Y. , Duffau, H. , Crivello, F. , Houdé, O. , … Tzourio-Mazoyer, N. (2006). Meta-analyzing left hemisphere language areas: Phonology, semantics, and sentence processing. NeuroImage, 30(4), 1414–1432. 10.1016/j.neuroimage.2005.11.002 16413796

[bib127] Vigneau, M. , Beaucousin, V. , Hervé, P. Y. , Jobard, G. , Petit, L. , Crivello, F. , … Tzourio-Mazoyer, N. (2011). What is right-hemisphere contribution to phonological, lexico-semantic, and sentence processing? Insights from a meta-analysis. NeuroImage, 54(1), 577–593. 10.1016/j.neuroimage.2010.07.036 20656040

[bib128] Vitali, P. , Abutalebi, J. , Tettamanti, M. , Danna, M. , Ansaldo, A.-I. , Perani, D. , … Cappa, S. F. (2007). Training-induced brain remapping in chronic aphasia: A pilot study. Neurorehabilitation and Neural Repair, 21, 152–160. 10.1177/1545968306294735 17312090

[bib129] Vitali, P. , Tettamanti, M. , Abutalebi, J. , Ansaldo, A. , Perani, D. , Cappa, S. F. , … Perani, D. (2010). Generalization of the effects of phonological training for anomia using structural equation modelling: A multiple single-case study. Neurocase, 16(2), 93–105. 10.1080/13554790903329117 19967599

[bib130] Wan, C. Y. , Zheng, X. , Marchina, S. , Norton, A. , Schlaug, G. , Laboratories, S. R. , & Deaconess, B. I. (2014). Intensive therapy induces contralateral white matter changes in chronic stroke patients with Broca’s aphasia. Brain and Language, 136, 1–7. 10.1016/j.bandl.2014.03.011 25041868 PMC4425280

[bib131] Wang, J. , Marchina, S. , Norton, A. C. , Wan, C. Y. , & Schlaug, G. (2013). Predicting speech fluency and naming abilities in aphasic patients. Frontiers in Human Neuroscience, 7(December), 1–13. 10.3389/fnhum.2013.00831 24339811 PMC3857577

[bib132] Warburton, E. , Price, C. J. , Swinburn, K. , & Wise, R. J. S. (1999). Mechanisms of recovery from aphasia: Evidence from positron emission tomography studies. Journal of Neurology, Neurosurgery, & Psychiatry, 66, 155–161. https://jnnp.bmj.com/content/66/2/155 10071093 10.1136/jnnp.66.2.155 PMC1736204

[bib133] Wierenga, C. E. , Maher, L. M. , Moore, A. B. , White, K. D. , McGregor, K. , Soltysik, D. A. , … Crosson, B. (2006). Neural substrates of syntactic mapping treatment: An fMRI study of two cases. Journal of the International Neuropsychological Society, 12, 132–146. https://europepmc.org/article/med/16433953 16433953 10.1017/S135561770606019X

[bib134] Witteman, J. , van Ijzendoorn, M. H. , van de Velde, D. , van Heuven, V. J. J. P. , & Schiller, N. O. (2011). The nature of hemispheric specialization for linguistic and emotional prosodic perception: A meta-analysis of the lesion literature. Neuropsychologia, 49(13), 3722–3738. 10.1016/j.neuropsychologia.2011.09.028 21964199

[bib135] Yarkoni, T. , Poldrack, R. A. , Nichols, T. E. , Van Essen, D. C. , & Wager, T. D. (2011). Large-scale automated synthesis of human functional neuroimaging data. Nature Methods, 8(8), 665–670. 10.1038/nmeth.1635 21706013 PMC3146590

[bib136] Yourganov, G. , Fridriksson, J. , Rorden, C. , Gleichgerrcht, E. , & Bonilha, L. (2016). Multivariate connectome-based symptom mapping in post-stroke patients: Networks supporting language and speech. The Journal of Neuroscience, 36(25), 6668–6679. 10.1523/JNEUROSCI.4396-15.2016 27335399 PMC4916245

