# Supplementary material for: A Review on Treatment-Related Brain Changes in Aphasia
Source: Neurobiol Lang (Camb). 2020 Oct 1;1(4):402–33. doi: 10.1162/nol_a_00019 (PMC10158631; doi:10.1162/nol_a_00019)
Supplement: Supplementary file 1 [file nol-1-4-402-s001.pdf]

Supplementary information

The language-processing network according to more elaborate language models

For semantic, phonological, orthographic and syntactic processing, we focused on the reviews on the functional neuroanatomy of language written by (Price, 2010, 2000, 2012), complemented with the meta-analysis of Jobard, Crivello, & Tzourio-Mazoyer (2003) for orthographic processing and the meta-analysis of Vigneau et al. (2006) for syntactic processing. Much less research has been conducted to find the neural correlates of rhythmic-melodic processing. In this study, the rhythmic-melodic aspect we are interested in, is linguistic prosody (compared to emotional prosody) and, more specifically, the use of intonation to make syntactic distinctions, such as the distinction between declarative and interrogative sentences. This is because as in Melodic Intonation Therapy (MIT), frequently provided to PWA, it involves slowly changing (1-2 Hz, Giraud & Poeppel (2012)) sentence-level intonation patterns. In MIT, the prosodic speech pattern of normal spoken sentences is transformed into two-pitched melodic intonation patterns (singing), accompanied by rhythmic syllable tapping (Schlaug et al., 2009). Meyer, Alter, & Friederici (2003) and Kreitewolf, Friederici, & von Kriegstein (2014) specifically focussed on syntactic prosody, which is why we chose to focus on the neural correlates identified in these studies as neural correlates of rhythmic-melodic processing inherent to language.

**Linguistic domain**	**Brain region**	**Function (within the linguistic component)**
Semantics	SFG_{med}	Amodal semantic processing/retrieval/selection dependent on semantic context, semantic decisions and control
	MFG	Word retrieval from semantics for speech production
	IFG_{tri}	Semantic processing of spoken words, semantic selection/(word) retrieval/decisions/association
	IFG_{orb}	Amodal semantic selection/retrieval/processing/control

	AG	Amodal semantic word retrieval/processing, sentence comprehension, crossmodal semantic integration/prediction
	precuneus	Semantic processing of spoken words, sentence and narrative comprehension, updating story representations
	midCING	Narrative language comprehension
	pCING	Amodal semantic processing, updating story representations
	aSTG	Semantic integration
	pMTG	Amodal semantic processing of words and sentences, semantic integration, word retrieval from semantics
	temporal pole	Semantic processing of spoken words, amodal sentence and narrative comprehension, semantic associations
	ITG	Amodal semantic processing of words and sentences, semantic associations, word retrieval from semantics
	fusiform/occipito-temporal	Amodal semantic processing, word retrieval from semantics, linking visual forms with semantics, accessing semantics from visual forms
	hippocampus	Amodal semantic processing (left), integration and consolidation of semantic concepts (right)
	cerebellum	Word retrieval from semantics, word acquisition
Phonology	IFG_{op}	Auditory verbal WM, (covert) articulatory planning, sequencing subsequent motor activity
	insula	Articulatory planning and coordination
	PMA	Vocal production, action selection/planning/initiation/execution/control, inner speech
	(pre)SMA	Articulation, sequencing motor plans/motor execution, inner speech, motor timing/control
	precentral	Motor execution and control (articulation)
	vSMG	Subvocal articulation, auditory self-monitoring, Auditory verbal WM, auditory expectations
	pSTS/STG	Auditory processing/feedback/imagery, phonemic processing, word retrieval from phonology
	pITG	Phonological retrieval (together with insula)
	cerebellum	Articulation, working memory, auditory self-monitoring, motor planning/timing/control
	thalamus	Articulation

	basal ganglia	Motor initiation/execution/timing
Orthography	IFG_{tri}	Semantic reading route
	IFG_{op}/precentral	Phonological reading route
	vSMG/AG	Phonological reading route
	precuneus	Visual imagery
	STS/STG	Phonological reading route
	pMTG/pITG	Semantic reading route
	pFusiform/occipito-temporal*	Local visual feature processing, early visual processing of sublexical forms, semantic reading route
	LOC/cuneus	Visual processing
	medLING*	Global visual feature processing
	calcarine	Early visual processing and visual imagery
Syntax	SFG	Syntactic processing during language comprehension
	pMFG	Syntactic WM
	$IFG_{op/tri}$	Hierarchical organization/sequencing of sequential events (such as words in sentences), auditory verbal WM
	insula	Syntactic processing during speech comprehension
	aSTS/STG	Multimodal sentence comprehension
	pMTG/STS	Multimodal sentence comprehension, syntactic WM
	temporal pole	Early syntactic processing, integration of syntactic and semantic information, organizing words into constituents
	basal ganglia	Late syntactic processing
Rhythmic-melodic processing	MFG/insula/FOP	Task effort? (R>L)
	precentral/IFG_{op}	Processing of slow prosodic modulations in lingual melodies (e.g. sentence intonation contour) (R>L)
	SMG	Working memory (covert rehearsal of pitch contours) (L>R)
	Heschl/pSTG/PT	Early auditory processing (R>L)
	ATL/PP	Morphosyntactic processing, integrating semantic and syntactic information during sentence comprehension (R>L)
	cerebellum	Sensory processes such as pitch discrimination (L>R)

Table 1 The different brain regions assumed to be involved in the different aspects of language and their specific contribution, according to more elaborate models of linguistic processing (see

text for further details). *Legend* SFG/MFG/IFG = superior/middle/inferior frontal gyrus, op = opercular, tri = triangular, orb = orbital, med = medial, SMA = supplementary motor area, FOP = frontal operculum, SMG = supramarginal gyrus, AG = angular gyrus, PT/PP = planum temporale/polare, STG/MTG/ITG = superior/middle/inferior temporal gyrus, ATL = anterior temporale lobe, SOG/MOG/IOG = superior/middle/inferior occipital gyrus, LOC = lateral occipital complex, CING = cingulum, ant = anterior, mid = middle, post = posterior, med = medial, v = ventral. *According to Price & Devlin (2003), there is no area specialized in visual word form processing, the controversial visual word-form area in the medial extrastriate cortex, introduced by Cohen et al. (2000) and Dehaene, Le Clec'H, Poline, Le Bihan, & Cohen (2002), but this function is presumably fulfilled by the interaction between visual, semantic and phonological processing.

The rhythmic-melodic network

The figure below represents the rhythmic-melodic network. As we explain in the main text of the review, there is no Neurosynth meta-analysis available for the keyword “rhythmic-melodic”, nor for related keywords (“rhythm”/”rhythmic”, “melody”/”melodic”, “prosody”/”prosodic”). For Table 1 (above), we derived the neural correlates of rhythmic-melodic processing (on the sentence level) from the studies by Meyer et al. (2003) and Kreitewolf et al. (2014). For illustration purposes, in Figure 1, we provide the reader with a visualization of the brain regions that are responding to rhythmic-melodic elements that are inherent to language on the sentence-level. As the figure is only based on the converging results of the two aforementioned studies, it should not be directly compared with Figure 1 of the main text, in which association test maps integrating hundreds of studies are visualized. Information on lateralization of the brain responses in the different regions in the network (comparing rhythmic-melodic processing vs. speech processing) is given in Table 1.

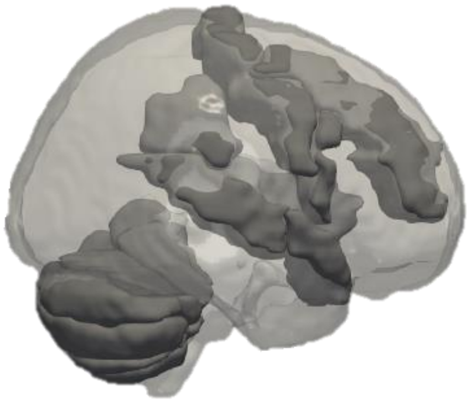

Figure 1 Regions involved in rhythmic-melodic processing at the sentence level, according to the converging results in the studies of Meyer et al. (2003) and Kreitewolf et al. (2014), shown in the right hemisphere. In Table 1, information on the relative contribution of the left hemisphere in rhythmic-melodic processing is provided. The figure was composed using Paraview software (version 5.4.1) following the guidelines specified in Madan (2015).

Details on the interventions provided in the studies included in the review

Interventions focusing on lexico-semantic processing

Approximately half of the included studies in PWA explore functional brain reorganization during a picture naming task after versus before engaging in a treatment on the lexical level. These interventions are specifically targeting anomia, an impairment that is common in PWA irrespective of the aphasic syndrome, and implies word retrieval failure. This is caused by deficits in retrieving the semantic aspects of a concept or in the access to the phonological form of a word, or a combination of both (Goodglass & Wingfield, 1997). Since each of both loci of breakdown in word retrieval results in a different kind of naming error, namely semantic or phonological paraphasias, two main therapy approaches exist, either focusing on semantic or phonological processing (van Hees et al., 2013). Nine of the included studies administer a treatment that specifically focuses on the semantic aspects of word retrieval (lexico-semantic training) (Abel, Weiller, Huber, & Willmes, 2014; Cornelissen et al., 2003; Fridriksson et al., 2007; Kiran, Meier, Kapse, & Glynn, 2015; Marcotte, Perlbarg, Marrelec, & Benali, 2013; Marcotte & Ansaldo, 2010; Sandberg et al., 2015; Van Hees, 2014a, 2014b).

The trainings described in these studies apply a semantic cueing (hierarchy) method (Fridriksson et al., 2007), Semantic Feature Analysis (SFA) (Boyle, 2004), a contextual priming technique (Cornelissen et al., 2003) or a method using abstractness as a mode of complexity (Sandberg et al., 2015) to improve naming. The underlying idea is that activation of the semantic features of a word increases the chance that this word will be retrieved due to spreading activation in the semantic network surrounding the representation of that word (Collins & Loftus, 1975). In this regard, SFA facilitates word retrieval using a matrix of cue words (e.g. group, use, action, properties) prompting the semantic features of the target word (Boyle, 2004), before and after which the word is named. Similarly, in a semantic cueing

hierarchy treatment semantic cues with increasing strength are given to the patient (Fridriksson et al., 2007). The contextual priming technique aims to improve naming based on principles of repetition- and semantic priming. The patients repeats the names of semantically related items several times and, at predefined moments, tries to name the object himself (Cornelissen et al., 2003). In contrast, the method focusing on generating abstract words used in Sandberg et al. (2015) is assumed to lead to generalization to less complex concrete words in the same semantic category (Sandberg et al., 2015).

Interventions focusing on lexico-phonological processing

An intervention on the phonological aspects of word retrieval (lexico-phonological training) is provided in 13 of the included studies (Abel et al., 2014; Brownsett et al., 2014; Fridriksson et al., 2006, 2007; Haldin et al., 2018; Leonard et al., 2015; Marcotte et al., 2018; Nardo et al., 2017; Rochon et al., 2010; Van Hees et al., 2014a, 2014b; Vitali et al., 2007, 2010). Similar to the lexico-semantic interventions, most trainings apply a phonological cueing (hierarchy) method or Phonological Components Analysis (PCA) (Van Hees et al., 2014a) to improve naming. PCA is the phonological variant of SFA, facilitating word retrieval using a matrix of cue words prompting phonological features of the target word (e.g. a rhyming word, the first sound, the number of syllables), before and after which the word is named (van Hees et al., 2013). In the cueing hierarchy treatment, cues are now phonological instead of semantic.

Other ”phon” studies applied treatment methods grounded on principles of spaced retrieval, errorless learning, massed practice (Fridriksson et al., 2006), auditory discrimination and repetition (Brownsett et al., 2014) and sensorimotor integration during speech (Haldin et al., 2018). Therapy methods grounded on spaced retrieval have originally been developed for persons with dementia to improve memory function. In this method, the time interval between

recall attempts of target information is gradually increased. Fridriksson et al. (2006) provide this therapy to PWA in a group setting to improve word retrieval. Patients had to repeat the target item three times, followed by three naming attempts based on a phonemic cue and a spontaneous naming attempt. Errorless learning was promoted by modelling the target item when naming failed to occur (Fridriksson et al., 2006). In addition, in the study of Brownsett et al. (2014) 16 PWA practiced with a home-based computer program consisting of five auditory discrimination and repetition tasks for 3 weeks. Finally, Haldin et al. (2018) investigated the Sensori-Motor Fusion intervention method in one patient with chronic non-fluent aphasia. The idea is that repetition of auditorily presented vowels, consonants and VCV sequences, while receiving visual information on target tongue and lip movements, will enhance the sensorimotor representation of the sounds in the brain.

Interventions focusing on semantic-phonological processing

Third, we considered studies that train both semantic and phonological aspects of word retrieval (phonological-semantic training). Several of the identified neuroimaging studies provide a semantic cueing hierarchy treatment alternated with a phonological cueing hierarchy treatment. However, they do not always differentiate between the two types of intervention when reporting neural activity changes or they additionally report general activation changes over the course of both treatments. Six such studies were identified (Abel et al., 2014, 2015; Fridriksson, 2010; Fridriksson, Richardson, Fillmore, & Cai, 2012b; Van Hees et al., 2014b, 2014c).

Interventions focusing on phonological-orthographic processing

Furthermore, two of the included studies provide a language intervention concentrated on phonological as well as orthographic aspects of language processing (Menke et al., 2009; Raboyeau et al., 2008). Lexical interventions based on a phonological cueing method regularly

provide graphemic cues to facilitate word retrieval, which is the case in the studies of Menke et al. (2009) and Raboyeau et al. (2008). This graphemic cue can be the written word form or the (two) first grapheme(s) of the target word. This method is grounded on the principle of associational learning, according to which the associations between the sound of the word, the written word form and the target picture will be strengthened when one is provided with multimodal cues (Menke et al., 2009).

Interventions focusing on morphosyntactic processing

In addition to semantic, phonological and orthographic problems, PWA frequently present various deficits in (morpho)syntax, including problems with word order, closed-class elements, thematic role assignment and action production. The latter deficit is considered as a word retrieval failure in the distinctive domain of verbs (vs. nouns). It is generally more evident in verbs with a more complex argument structure, hence tapping on syntactic aspects (Wierenga et al., 2006). Three of the included studies focused on the production and comprehension of verbs and sentences (syntactic-semantic processing) and are labelled with ”syntax” (Thompson, den Ouden, Bonakdarpour, Garibaldi, & Parrish, 2010; Thompson, Riley, Ouden, Meltzer-asscher, & Lukic, 2013; Wierenga et al., 2006).

The provided interventions either tap in on the comprehension and production of non-canonical sentences with the Treatment of Underlying Forms (TUF) (Thompson & Shapiro, 2005), used a syntactic mapping treatment (Wierenga et al., 2006) or applied a treatment focused on verb argument structure (Thompson et al., 2013). TUF applies a linguistic approach to the treatment of object-extracted wh-questions (‘Who did the thief chase?’), object-clefts (‘It was the artist who the thief chased.’) and object-relative clauses (‘The man saw the artist that the thief chased.’) (for review see Thompson & Shapiro, 2005). The syntactic mapping treatment focuses

on the production of semantically reversible active ('The boy is kissing the girl.') and passive ('The girl is kissed by the boy.') sentences. Finally, the treatment focused on verb argument structure deals with active sentences containing 3-argument verbs ('The boy **is giving** the *apple* to the *teacher*.'). The two latter methods both use syntactic cues to support thematic role assignment, such as therapeutic model, word cards with the verb and its arguments or a visual syntactic mapping template (Thompson et al., 2013; Wierenga et al., 2006).

Interventions focusing on rhythmic-melodic processing

Finally, five of the included neuroimaging studies apply rhythmic-melodic language interventions (Jungblut, Huber, Mais, & Schnitker, 2014; Schlaug, Marchina, & Norton, 2008, 2009; Tabei et al., 2016; Wan et al., 2014). Treatments tapping in on the rhythmic-melodic aspects of language have emerged after the discovery that people suffering from non-fluent aphasia with large left-hemisphere lesions were able to produce words while they were singing, but not while they were speaking (Hébert, Racette, Gagnon, & Peretz, 2003). Since singing is thought to rely more on the (non-damaged) right-hemispheric homologues of the well-known left-hemispheric language regions, Melodic Intonation Therapy (MIT) was invented to make use of this advantage (Schlaug et al., 2009). This treatment is hierarchically structured and transforms the prosodic speech pattern of normal spoken sentences into two-pitched melodic intonation patterns (singing), accompanied by rhythmic syllable tapping (Schlaug et al., 2009). This intensive and long lasting therapy is assumed to engage the damaged left-hemisphere through interhemispheric transfer (Al-janabi et al., 2014).

Another rhythmic-melodic voice training tapping in on the suprasegmental aspects of language is SIPARI, which was specifically developed for patients suffering from non-fluent aphasia accompanied by apraxia of speech. This treatment combines Singing, Intonation, Prosody,

breathing (Atmung in German), Rhythm and Improvisation (Jungblut et al., 2014). The initial focus is on vocal training of melodic speech segments assumed to be supported by the right hemisphere. Subsequently, the focus shifts to rhythmic chunking of these speech segments with different complexity levels to stimulate the left hemisphere. In summary, the singing voice unites linguistics, motor skills and cognition and is demonstrated to be very useful in non-fluent aphasia intervention, in which these three components are frequently disturbed (Jungblut et al., 2014).

Rationale behind the decision to include studies with shared participants

In total, 11 studies shared participants with one or two other studies. Nine of these studies were considered separately because they applied different, and mostly unrelated, analyses (see details of specific studies in point A below) or because there was only minimal overlap in participants and different interventions were considered (see details of specific studies in point B below). The studies reported by Abel, Weiller, Huber, & Willmes (2014) and Abel, Weiller, Huber, Willmes, & Specht (2015) were treated as one study because the same voxel-wise whole-brain contrast analysis has been reported in both studies.

A. The following studies examined the same participants, but used a different and unrelated analytic approach or imaging modality and were therefore considered separately:

- Van Hees et al. (2014a), Van Hees et al. (2014b) and Van Hees et al. (2014c)
    - Study 2014a: Task-based fMRI followed by a voxel-wise whole-brain contrast and regression analysis on treatment effects (picture naming vs. watching abstract pictures).
    - Study 2014b: Resting-state fMRI followed by functional connectivity analyses in language-related regions with amplitude of low frequency fluctuations.
    - Study 2014c: Diffusion-weighted imaging followed by region of interest based volumetric analysis (arcuate fasciculus and uncinate fasciculus).
- Vitali et al. (2007) and Vitali et al. (2010)
    - 2007: Task-based fMRI followed by a voxel-wise whole-brain contrast analysis (successfully trained picture naming vs. successfully untrained picture naming).
    - 2010: Region of interest based fMRI effective connectivity analysis with structural equation modeling.

B. Finally, the following studies share participants, but were still considered as separate studies for the following reasons:

- Fridriksson et al. (2006) and Fridriksson et al. (2007) share one patient. Because they investigated different treatments and because we cannot rule out overlap in any of the other studies, the results of both Fridriksson studies were considered separately.
    - 2006: Task-based fMRI followed by a voxel-wise whole-brain contrast analysis; anomia treatment based on principles of spaced retrieval, errorless learning and massed practice.
    - 2007: Task-based fMRI followed by a voxel-wise whole-brain and region of interest contrast analysis; anomia treatment based on a phonological or semantic cueing hierarchy (alternating).
- Fridriksson (2010) and Fridriksson et al. (2012b) share 26 out of 30 patients and applied the same treatment, but used univariate vs. multivariate analysis. Because the results of the 2012b study are not presented in anatomical regions of interest, but either reflect changes in individually defined “residual naming areas” or “perilesional areas”, they could not be included in Table 3 and Figures 4 and 5. However, the study is discussed in the review.
    - 2010: Task-based fMRI followed by voxel-wise whole-brain regression analysis and voxel-based lesion-symptom mapping (univariate analysis).
    - 2012b: Task-based fMRI followed by volume of interest based multiple linear regression analysis (multivariate analysis).

References

Abel, S., Weiller, C., Huber, W., & Willmes, K. (2014). Neural underpinnings for model-oriented therapy of aphasic word production. *Neuropsychologia*, *57*(1), 154–165. https://doi.org/10.1016/j.neuropsychologia.2014.03.010

Abel, S., Weiller, C., Huber, W., Willmes, K., & Specht, K. (2015). Therapy-induced brain reorganization patterns in aphasia. *Brain*, *138*(4), 1097–1112. https://doi.org/10.1093/brain/awv022

Adair, J. C., Nadeau, S. E., Conway, T. W., Gonzalez-Rothi, L. J., Heilman, P. C., Green, I. A., & Heilman, K. M. (2000). Alterations in the functional anatomy of reading induced by rehabilitation of an alexic patient. *Neuropsychiatry, Neuropsychology and Behavioral Neurology*, *13*(4), 303–311.

Boyle, M. (2004). Semantic feature analysis rreatment for anomia in two fluent aphasia syndromes. *American Journal of Speech-Language Pathology*, *13*(August), 236–249. https://doi.org/10.1044/1058-0360(2004/025)

Breier, J. I., Randle, S., Maher, L. M., & Papanicolaou, A. C. (2010). Changes in maps of language activity activation following melodic intonation therapy using magnetoencephalography: Two case studies. *Journal of Clinical and Experimental Neuropsychology*, *32*(3), 309–314. https://doi.org/10.1080/13803390903029293

Brownsett, S. L. E., Warren, J. E., Geranmayeh, F., Woodhead, Z., Leech, R., & Wise, R. J. S. (2014). Cognitive control and its impact on recovery from aphasic stroke. *Brain: A Journal of Neurology*, *137*(Pt 1), 242–254. https://doi.org/10.1093/brain/awt289

Cohen, L., Dehaene, S., Naccache, L., Lehéricy, S., Dehaene-Lambertz, G., Hénaff, M.-A., & Michel, F. (2000). The visual word form area: spatial and temporal characterization of an initial stage of reading in normal subjects and posterior split-brain patients. *Brain: A Journal of Neurology*, *123*, 291–307.

Collins, A. M., & Loftus, E. F. (1975). A spreading-activation theory of semantic processing. *Psychological Review*, *82*(6), 407–428. https://doi.org/10.1037/0033-295X.82.6.407

Cornelissen, K., Laine, M., Tarkiainen, A., Järvensivu, T., Martin, N., & Salmelin, R. (2003). Adult Brain Plasticity Elicited by Anomia Treatment. *Journal of Cognitive Neuroscience*, *15*(3), 444–461. https://doi.org/10.1162/089892903321593153

Davis, C. H., & Harrington, G. (2006). Intensive semantic intervention in fluent aphasia: A pilot study with fMRI. *Aphasiology*, *20*(1), 59–83. https://doi.org/10.1080/02687030500331841

Dehaene, S., Le Clec'H, G., Poline, J.-B., Le Bihan, D., & Cohen, L. (2002). The visual word form area: a prelexical representation of visual words in the fusiform gyrus. *Neuroreport*, *13*(3), 321–325. https://doi.org/10.1097/00001756-200203040-00015

Fridriksson, J. (2010). Preservation and modulation of specific left hemisphere regions is vital for treated recovery from anomia in stroke. *Journal of Neurophysiology*, *30*(35), 11558–11564. https://doi.org/10.1523/JNEUROSCI.2227-10.2010.

Fridriksson, J., Morrow-Odom, L., Moser, D., Fridriksson, A., & Baylis, G. (2006). Neural recruitment associated with anomia treatment in aphasia. *NeuroImage*, *32*(3), 1403–1412. https://doi.org/10.1016/j.neuroimage.2006.04.194

Fridriksson, J., Moser, D., Bonilha, L., Morrow-odom, K. L., Shaw, H., Fridriksson, A., Baylis, G. C., & Rorden, C. (2007). Neural correlates of phonological and semantic-based anomia treatment in aphasia. *Neuropsychologia*, *45*(8), 1812–1822. https://doi.org/10.1016/j.neuropsychologia.2006.12.017

Fridriksson, J., Richardson, J. D., Fillmore, P., & Cai, B. (2012b). Left hemisphere plasticity and aphasia recovery. *NeuroImage*, *60*(2), 854–863. https://doi.org/10.1016/j.neuroimage.2011.12.057

Giraud, A., & Poeppel, D. (2012). Cortical oscillations and speech processing: emerging

computational principles and operations. *Nature Neuroscience*, *15*(4), 511–517. https://doi.org/10.1038/nn.3063.

Goodglass, H., & Wingfield, A. (1997). *Anomia: Neuroanatomical and Cognitive Correlates* (H. Goodglass & A. Wingfield (eds.)). San Diego, California: Academic Press.

Haldin, C., Acher, A., Kauffmann, L., Hueber, T., Cousin, E., Badin, P., Perrier, P., Fabre, D., Perennou, D., Detante, O., Jaillard, A., Lœvenbruck, H., & Baciu, M. (2018). Speech recovery and language plasticity can be facilitated by Sensori-Motor Fusion training in chronic non-fluent aphasia. A case report study. *Clinical Linguistics and Phonetics, 32*(7), 595–621. https://doi.org/10.1080/02699206.2017.1402090

Jobard, G., Crivello, F., & Tzourio-Mazoyer, N. (2003). Evaluation of the dual route theory of reading: a metanalysis of 35 neuroimaging studies. *NeuroImage*, *20*(2), 693–712. https://doi.org/10.1016/S1053-8119(03)00343-4

Jungblut, M., Huber, W., Mais, C., & Schnitker, R. (2014). Paving the Way for Speech : Voice-Training-Induced Plasticity in Chronic Aphasia and Apraxia of Speech — Three Single Cases. *Neural Plasticity, 2014,* 1–14. https://doi.org/http://dx.doi.org/10.1155/2014/841982

Kiran, S., Meier, E. L., Kapse, K. J., & Glynn, P. A. (2015). Changes in task-based effective connectivity in language networks following rehabilitation in post-stroke patients with aphasia. *Frontiers in Human Neuroscience,* *9*(June), 1–20. https://doi.org/10.3389/fnhum.2015.00316

Kreitewolf, J., Friederici, A. D., & von Kriegstein, K. (2014). Hemispheric lateralization of linguistic prosody recognition in comparison to speech and speaker recognition. *NeuroImage*, *102*(P2), 332–344. https://doi.org/10.1016/j.neuroimage.2014.07.038

Leonard, C., Laird, L., Burianová, H., Graham, S., Grady, C., Simic, T., & Rochon, E. (2015). Behavioural and neural changes after a “choice” therapy for naming deficits in aphasia:

preliminary findings. *Aphasiology, 29*(4), 506–525. https://doi.org/10.1080/02687038.2014.971099

Madan, C. R. (2015). Creating 3D visualizations of MRI data: A brief guide. *F1000Research*, 466, 1–13. https://doi.org/10.12688/f1000research.6838.1

Marcotte, K., Laird, L., Bitan, T., Meltzer, J. A., Graham, S. J., Leonard, C., & Rochon, E. (2018). Therapy-induced neuroplasticity in chronic aphasia after phonological component analysis: A matter of intensity. *Frontiers in Neurology*, *9*(APR), 1–7. https://doi.org/10.3389/fneur.2018.00225

Marcotte, Karine, Adrover-Roig, D., Damien, B., de Préaumont, M., Généreux, S., Hubert, M., & Ansaldo, A. I. (2012). Therapy-induced neuroplasticity in chronic aphasia. *Neuropsychologia*, *50*(8), 1776–1786. https://doi.org/10.1016/j.neuropsychologia.2012.04.001

Marcotte, Karine, & Ansaldo, A. I. (2010). The neural correlates of semantic feature analysis in chronic aphasia: Discordant patterns according to the etiology. *Seminars in Speech and Language*, *31*(1), 52–63. https://doi.org/10.1055/s-0029-1244953

Meinzer, M., Mohammadi, S., Kugel, H., Schiffbauer, H., Flöel, A., Albers, J., Kramer, K., Menke, R., Baumgärtner, A., Knecht, S., Breitenstein, C., & Deppe, M. (2010). Integrity of the hippocampus and surrounding white matter is correlated with language training success in aphasia. *NeuroImage*, *53*(1), 283–290. https://doi.org/10.1016/j.neuroimage.2010.06.004

Menke, R., Meinzer, M., Kugel, H., Deppe, M., Baumgärtner, A., Schiffbauer, H., Thomas, M., Kramer, K., Lohmann, H., Flöel, A., Knecht, S., & Breitenstein, C. (2009). Imaging short- and long-term training success in chronic aphasia. *BMC Neuroscience*, *10*, 118. https://doi.org/10.1186/1471-2202-10-118

Meyer, M., Alter, K., & Friederici, A. (2003). Functional MR imaging exposes differential brain

responses to syntax and prosody during auditory sentence comprehension. *Journal of Neurolinguistics*, *16*(4–5), 277–300. https://doi.org/10.1016/S0911-6044(03)00026-5

Nardo, D., Holland, R., Leff, A. P., Price, C. J., & Crinion, J. T. (2017). Less is more: Neural mechanisms underlying anomia treatment in chronic aphasic patients. *Brain*, *140*(11), 3039–3054. https://doi.org/10.1093/brain/awx234

Norton, A., Zipse, L., Marchina, S., & Schlaug, G. (2009). Melodic intonation therapy: Shared insights on how it is done and why it might help. *Annals of the New York Academy of Sciences*, *1169*, 431–436. https://doi.org/10.1111/j.1749-6632.2009.04859.x

Price, C. J. (2000). The anatomy of language: contributions from functional neuroimaging. *Journal of Anatomy*, *197*(3), 335–359. https://doi.org/10.1046/j.1469-7580.2000.19730335.x

Price, C. J. (2012). A review and synthesis of the first 20 years of PET and fMRI studies of heard speech, spoken language and reading. *NeuroImage*, *62*(2), 816–847. https://doi.org/10.1016/j.neuroimage.2012.04.062

Price, C. J., & Devlin, J. T. (2003). The myth of the visual word form area. *NeuroImage, 19*(3), 473–481. https://doi.org/10.1016/S1053-8119(03)00084-3

Price, C. J., Seghier, M. L., & Leff, A. P. (2010). Predicting language outcome and recovery after stroke : the PLORAS system. *Nature Reviews Neurology*, *6*(4), 202–210. https://doi.org/10.1038/nrneurol.2010.15

Raboyeau, G., De Boissezon, X., Marie, N., Balduyck, S., Puel, M., Bézy, C., Démonet, J. F., & Cardebat, D. (2008). Right hemisphere activation in recovery from aphasia: Lesion effect or function recruitment ? *Neurology, 70,* 290–298. https://doi.org/10.1212/01.wnl.0000287115.85956.87

Rochon, E., Leonard, C., Burianova, H., Laird, L., Soros, P., Graham, S., & Grady, C. (2010). Neural changes after phonological treatment for anomia: An fMRI study. *Brain and*

Language, *114*(3), 164–179. https://doi.org/10.1016/j.bandl.2010.05.005

Schlaug, G., Marchina, S., & Norton, A. (2008). From Singing to Speaking: Why Singing May Lead to Recovery of Expressive Language Function in Patients with Broca's Aphasia. *Music Percept.*, *25*(4), 315–323. https://doi.org/10.1525/MP.2008.25.4.315.From

Schlaug, G., Marchina, S., & Norton, A. (2009). Evidence for Plasticity in White-Matter Tracts of Patients with Chronic Broca's Aphasia Undergoing Intense Intonation-based Speech Therapy. *Annals of the New York Academy of Sciences*, *1169*, 385–394. https://doi.org/10.1111/j.1749-6632.2009.04587.x

Tabei, K. I., Satoh, M., Nakano, C., Ito, A., Shimoji, Y., Kida, H., Sakuma, H., & Tomimoto, H. (2016). Improved neural processing efficiency in a chronic aphasia patient following melodic intonation therapy: A neuropsychological and functional MRI study. *Frontiers in Neurology*, *7*(SEP), 1–6. https://doi.org/10.3389/fneur.2016.00148

Thompson, C. K. (2000). Neuroplasticity: evidence from aphasia. *Journal of Communication Disorders*, *33*(847), 357–366.

Thompson, C. K., den Ouden, D.-B., Bonakdarpour, B., Garibaldi, K., & Parrish, T. B. (2010). Neural plasticity and treatment-induced recovery of sentence processing in agrammatism. *Neuropsychologia*, *48*(11), 3211–3227. https://doi.org/10.1038/jid.2014.371

Thompson, C. K., Fix, S. C., Gitelman, D. R., Parrish, T. B., & Mesulam, M.-M. (2000). FMRI Studies of Agrammatic Sentence Comprehension Before and After Treatment. *Brain and Language*, *74*(3), 387–391. https://doi.org/10.1006/brln.2000.2364

Thompson, C. K., Riley, E. A., den Ouden, D.-B., Meltzer-asscher, A., & Lukic, S. (2013). Training verb argument structure production in agrammatic aphasia: Behavioral and neural recovery patterns. *CORTEX*, *49*(9), 2358–2376. https://doi.org/10.1016/j.cortex.2013.02.003

Thompson, C. K., & Shapiro, L. P. (2005). Treating agrammatic aphasia within a linguistic

framework: Treatment of Underlying Forms. *Aphasiology*, *19*(10–11), 1021–1036.

van Hees, S., Angwin, A., McMahon, K., & Copland, D. (2013). A comparison of semantic feature analysis and phonological components analysis for the treatment of naming impairments in aphasia. *Neuropsychological Rehabilitation*, *23*(1), 102–132. https://doi.org/10.1080/09602011.2012.726201

Van Hees, S., McMahon, K., Angwin, A., de Zubicaray, G., & Copland, D. A. (2014a). Neural activity associated with semantic versus phonological anomia treatments in aphasia. *Brain and Language*, *129*(1), 47–57. https://doi.org/10.1016/j.bandl.2013.12.004

Van Hees, S., Mcmahon, K., Angwin, A., de Zubicaray, G., Read, S., & Copland, D. A. (2014b). A functional MRI study of the relationship between naming treatment outcomes and resting state functional connectivity in post-stroke aphasia. *Human Brain Mapping*, *35*(8), 3919–3931. https://doi.org/10.1002/hbm.22448

Van Hees, S., Mcmahon, K., Angwin, A., Zubicaray, G. De, Read, S., & Copland, D. A. (2014c). Changes in White Matter Connectivity Following Therapy for Anomia Post stroke. *Neurorehabilitation and Neural Repair*, *28*(4), 325 –334. https://doi.org/10.1177/1545968313508654

Vigneau, M., Beaucousin, V., Hervé, P. Y., Duffau, H., Crivello, F., Houdé, O., Mazoyer, B., & Tzourio-Mazoyer, N. (2006). Meta-analyzing left hemisphere language areas: Phonology, semantics, and sentence processing. *NeuroImage*, *30*(4), 1414–1432. https://doi.org/10.1016/j.neuroimage.2005.11.002

Vitali, P, Tettamanti, M., Abutalebi, J., Ansaldo, A., Perani, D., Cappa, S. F., Joanette, Y., Tettamanti, M., Abutalebi, J., Ansaldo, A., & Perani, D. (2010). Generalization of the effects of phonological training for anomia using structural equation modelling : A multiple single-case study. *Neurocase*, *16*(2), 93–105. https://doi.org/10.1080/13554790903329117

Vitali, Paolo, Abutalebi, J., Tettamanti, M., Danna, M., Ansaldo, A., Perani, D., Joanette, Y., & Cappa, S. F. (2007). Training-Induced Brain Remapping in Chronic Aphasia: A Pilot Study. *Neurorehabilitation and Neural Repair*, *21*, 152–160. https://doi.org/10.1177/1545968306294735

Wan, C. Y., Zheng, X., Marchina, S., Norton, A., Schlaug, G., Laboratories, S. R., & Deaconess, B. I. (2014). Intensive therapy induces contralateral white matter changes in chronic stroke patients with Broca's aphasia. *Brain and Language*, *136*, 1–7. https://doi.org/10.1016/j.bandl.2014.03.011.

Wierenga, C. E., Maher, L. M., Moore, A. B., White, K. D., Mcgregor, K., Soltysik, D. A., Peck, K. K., Gopinath, K. S., Singletary, F., Gonzalez-rothi, L. J., Briggs, R. W., & Crosson, B. (2006). Neural substrates of syntactic mapping treatment: An fMRI study of two cases. *Journal of the International Neuropsychological Society*, *12*, 132–146.
